# Glutaminase 1 expression in colorectal cancer cells is induced by hypoxia and required for tumor growth, invasion, and metastatic colonization

**DOI:** 10.1038/s41419-018-1291-5

**Published:** 2019-01-17

**Authors:** Lisha Xiang, Jun Mou, Bin Shao, Yuquan Wei, Houjie Liang, Naoharu Takano, Gregg L. Semenza, Ganfeng Xie

**Affiliations:** 1 Institute of Drug Clinical Trial, State Key Laboratory of Biotherapy and Cancer Center, West China Hospital, Sichuan University, Chengdu, 610041 China; 2 Department of Oncology and Southwest Cancer Center, Southwest Hospital, Third Military Medical University (Army Medical University), Chongqing, China; 3 Department of biochemistry, Tokyo medical University, Tokyo, Japan; 4 Vascular Program, Institute for Cell Engineering, Johns Hopkins University School of Medicine, Baltimore, MD 21205 USA; 5 McKusick-Nathans Institute of Genetic Medicine, Johns Hopkins University School of Medicine, Baltimore, MD 21205 USA; 6 Departments of Pediatrics, Medicine, Oncology, Radiation Oncology, and Biological Chemistry, Johns Hopkins University School of Medicine, Baltimore, MD 21205 USA

## Abstract

Cancer cells re-program their metabolic machinery to meet the requirements of malignant transformation and progression. Glutaminase 1 (GLS1) was traditionally known as a mitochondrial enzyme that hydrolyzes glutamine into glutamate and fuels rapid proliferation of cancer cells. However, emerging evidence has now revealed that GLS1 might be a novel oncogene involved in tumorigenesis and progression of human cancers. In this study, we sought to determine whether GLS1 implicated in invasion and metastasis of colorectal carcinoma, and its underlying molecular mechanism. By analyzing a large set of clinical data from online datasets, we found that GLS1 is overexpressed in cancers compared with adjacent normal tissues, and associated with increased patient mortality. Immunohistochemical analysis of GLS1 staining showed that high GLS1 expression is significantly correlated with lymph node metastasis and advanced clinical stage in colorectal cancer patients. To investigate the underlying mechanism, we analyzed the Cancer Genome Atlas database and found that GLS1 mRNA expression is associated with a hypoxia signature, which is correlated with an increased risk of metastasis and mortality. Furthermore, reduced oxygen availability increases GLS1 mRNA and protein expression, due to transcriptional activation by hypoxia-inducible factor 1. GLS1 expression in colorectal cancer cells is required for hypoxia-induced migration and invasion in vitro and for tumor growth and metastatic colonization in vivo.

## Introduction

Reprogramming of cancer cell metabolism leads to increased aerobic glycolysis (Warburg effect), which ultimately fuels the tricarboxylic acid (TCA) cycle and provides energy and biomass for rapid proliferating cells^1^. In addition to glucose metabolism, cancer cells rely on increased glutamine metabolism to maintain a functioning TCA cycle. The conversion of glutamine to glutamate is catalyzed by mitochondrial glutaminase activity. In malignancies, elevated glutaminolysis provides a substrate for macromolecule biosynthesis and ATP generation^2^. Two genes encode glutaminase in mammalian cells: *Glutaminase 1* (*GLS1*) is located on chromosome 2 and encodes the kidney-type isoenzyme (KGA), whereas *GLS2* is located on chromosome 12 and encodes the liver-type isoform (LGA)^3^.

Recent studies have reported the involvement of glutaminase in tumor cell proliferation^4^, autophagy^5^, signal transduction^6^, and radioresistance^7^. However, glutamine metabolism has been implicated in tumor metastasis^8^. Interestingly, targeting glutamine metabolism by a glutamine analog (DON, 6-diazo-5-oxo-l-norleucine), which is also an inhibitor of phosphate-activated glutaminase^9^, inhibits systemic metastasis in the VM-M3 murine tumor model^8^. These data suggest that GLS1 activity may promote metastasis, which is the major cause of cancer patient mortality. To test this hypothesis, we analyzed public datasets and tumor tissue microarrays from colorectal carcinoma patients. Our results show that GLS1 activity is significantly correlated with advanced clinical stage and lymph node metastasis in colorectal cancer patients, as well as patient mortality.

To investigate the underlying regulatory mechanism, we searched for correlations between gene signatures and GLS1, which revealed that GLS1 mRNA expression was correlated with multiple genes upregulated under hypoxic conditions. In multiple types of advanced human cancer, the presence of intratumoral hypoxia is a characteristic property, and has been identified as an adverse prognostic factor for patient outcome^10^. Cells adapt to hypoxia through the activity of the hypoxia-inducible factors (HIFs), which are transcriptional activators that regulate the expression of thousands of target genes^10,11^. HIFs are heterodimers composed of an O_2_-regulated HIF-1α or HIF-2α subunit and a constitutively expressed HIF-1β subunit^12^. In normoxic cells, HIF-1α is subject to prolyl and asparaginyl hydroxylation, ubiquitination, and proteasomal degradation^13,14^. The prolyl and asparaginyl hydroxylation reactions are inhibited in hypoxic cells, leading to rapid accumulation of HIF-1α, dimerization with HIF-1β, binding to the consensus DNA sequence 5′-RCGTG-3′ within hypoxia response elements (HREs) located in target genes, and transcriptional activation^15^.

HIFs activate the transcription of target genes that are involved in many crucial aspects of cancer biology including angiogenesis^16^, stem cell maintenance^17,18^, autocrine growth factor signaling^19^, epithelial–mesenchymal transition^20^, chemo- and radioresistance^21,22^, invasion^23^, and metastasis^24–26^. HIF-1 also regulates many metabolic processes in cancer cells. For example, HIF-1 mediates the expression of genes encoding glucose transporters (*GLUT1, GLUT3*) and glycolytic enzymes (*ENO1, ALDOA, GAPDH, PFKL, PGK1, PKM2, HK1, HK2, LDHA*) that convert glucose to lactate^27^. Moreover, HIF-1 regulates pyruvate dehydrogenase kinase 1 (*PDK1*), which inhibits mitochondrial oxidative metabolism^28^. HIFs coordinately regulate three genes encoding enzymes of the serine synthesis pathway in breast cancer cells^29^. HIF-1 was also reported to regulate the expression of transketolase enzymes (*TKT, TKTL2*) of the pentose phosphate pathway in a leukemia-like cell line^30^. In the present study, we demonstrate that HIF-1 regulates glutamine metabolism by activating the expression of the *GLS1* gene encoding mitochondrial GLS1 in colorectal carcinoma, which is required for hypoxia-induced cancer cell migration, invasion, and metastatic colonization.

## Results

### High GLS1 expression is associated with poor prognosis in human cancers

**Fig. 1:**
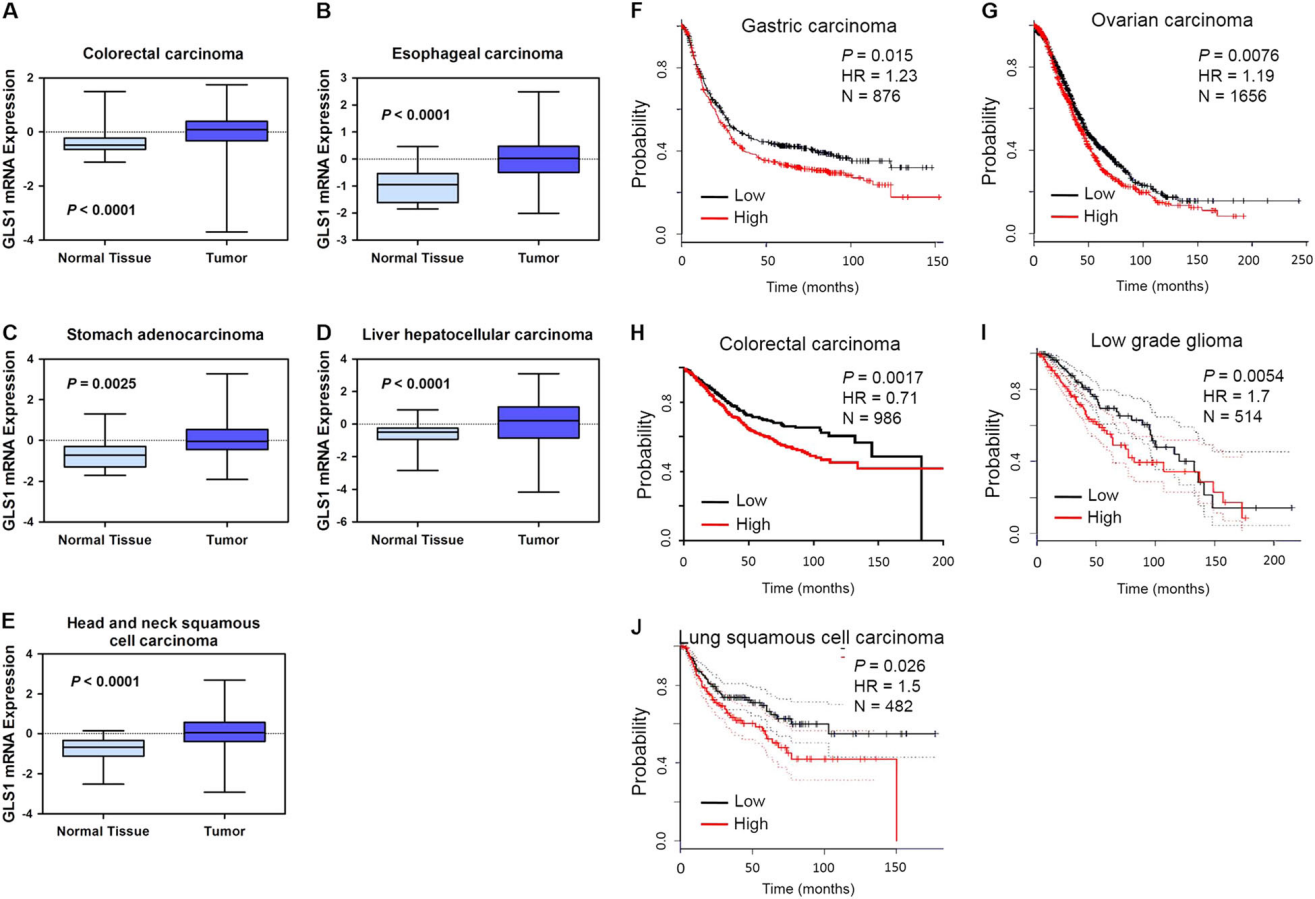
Glutaminase 1 (GLS1) high expression is associated with poor prognosis in human cancers. Relative levels of GLS1 mRNA from microarray analysis (normalized log2 ratios) of primary tumor samples relative to adjacent normal tissue from cancer patients (the Cancer Genome Atlas (TCGA) database) are shown. **a** Light blue, samples from normal colorectal tissue (*n* = 50); Dark blue, samples from colorectal adenocarcinomas (*n* = 383), *p* < 0.0001. **b** Light blue, samples from normal esophageal tissue (*n* = 13); Dark blue, samples from esophageal carcinomas (*n* = 185), *p* < 0.0001. **c** Light blue, samples from normal stomach tissue (*n* = 37); Dark blue, samples from stomach adenocarcinomas (*n* = 384), *p* = 0.0025. **d** Light blue, samples from normal liver tissue (*n* = 50); Dark blue, samples from liver hepatocellular carcinomas (*n* = 373), *p* < 0.0001. **e** Light blue, samples from normal head and neck tissue (*n* = 43); Dark blue, samples from head and neck squamous cell carcinomas (*n* = 521), *p* < 0.0001. Mann–Whitney *U*-test or analysis of variance (ANOVA) followed by Bonferroni post-test for multiple comparisons was used to determine *p*-values. **f**–**j** Kaplan–Meier curves were constructed to analyze the association of GLS1 mRNA levels in the primary tumor with the probability of overall survival (**f**, gastric carcinoma, *n* = 876, *p* = 0.015; **g**, ovarian carcinoma, *n* = 1656, *p* = 0.0076; **h** colorectal carcinoma, *n* = 986, *p* = 0.0017; **i**, low-grade glioma, *n* = 514, *p* = 0.0054) or disease free survival (**j**, lung squamous cell carcinoma, *n* = 482, *p* = 0.026) using the KM plotter, NCBI Gene Expression Omnibus or GEPIA database. Low = patients with GLS1 mRNA levels less than the median. High = patients with GLS1 mRNA levels greater than the median. Statistical analysis was performed using log-rank tests

To investigate whether GLS1 expression has clinical significance in human cancer, we compared *GLS1* gene expression in many different types of human cancer and their adjacent normal tissue using the Cancer Genome Atlas (TCGA) database (https://genome-cancer.ucsc.edu). Analysis of representative datasets of different human cancers revealed that GLS1 mRNA levels were significantly greater in human cancer tissue (colorectal, esophageal, gastric, hepatocellular, and head and neck squamous cell carcinoma) than in the respective adjacent normal tissues (Fig. 1a–e). The results were similar when we interrogated the Oncomine database (www.oncomine.org) for *GLS1* expression in human glioblastoma (*p* = 6.33e–14), breast cancer (*p* = 5.11e–5), and colorectal cancer (*p* = 3.48e–11) versus their adjacent normal tissue (Fig. S1A–C). These results indicate that *GLS1* is preferentially expressed in many human cancers compared with normal tissue.

We analyzed the relationship between *GLS1* expression and patient survival using public databases. Kaplan–Meier plots (http://www.kmplot.com) of 876 gastric cancer patients revealed that gastric cancers with high expression (above the median) of GLS1 mRNA were associated with significantly decreased patient survival over 150 months as compared with cancers with low expression of GLS1 (*p* = 0.015) (Fig. 1f). Increased GLS1 mRNA expression was also associated with decreased survival in 1656 ovarian cancer patients (*p* = 0.0076) (Fig. 1g), and NCBI Gene Expression Omnibus data (http://www.ncbi.nlm.nih.gov/geo/) for 986 cases of colorectal cancer (*p* = 0.0017) (Fig. 1h). Analysis of 514 low-grade glioma patients (Fig. 1i, *p* = 0.0054) and 482 lung squamous cell cancer patients (Fig. 1j, *p* = 0.026) in GEPIA database (http://gepia.cancer-pku.cn) revealed that GLS1 mRNA levels above the median were significantly associated with decreased patient survival in low-grade glioma and squamous cell lung cancer. Taken together, results obtained from multiple databases indicate that GLS1 is a potential prognostic biomarker for multiple types of human cancers.

### The high expression of GLS1 in colorectal carcinoma is significantly correlated with the presence of lymph node metastasis and advanced clinical stage

**Fig. 2:**
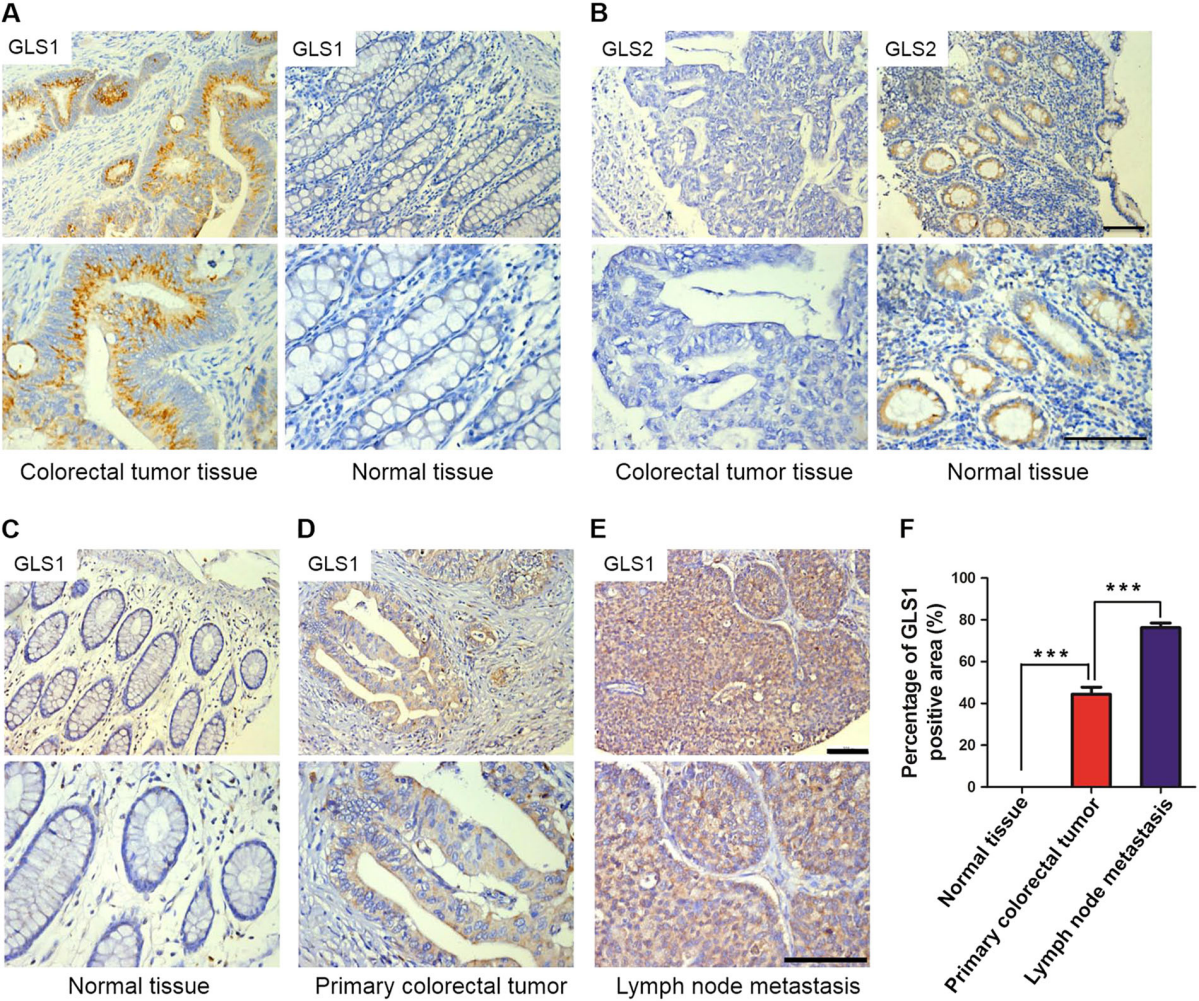
Immunohistochemical staining of glutaminase 1 (GLS1) in human colorectal carcinoma and adjacent normal colorectal tissue. **a**, **b** Representative images of GLS1 (**a**) and GLS2 (**b**) expression in tissue microarrays containing 39 cases of colorectal adenocarcinoma and 15 cases of normal colorectal tissue are shown. The bottom panel shows higher magnification of GLS1 and GLS2 staining (brown). **c**–**e** Representative images of GLS1 in tissue microarrays containing tumor samples from 85 colorectal cancer patients (24 patients with the presence of lymph node metastasis) are shown, respectively. **c** Adjacent normal colorectal tissue; **d** Primary colorectal tumor tissue; **e** Lymph node metastasis. The bottom panel shows higher magnification of GLS1 staining. Scale bar = 200 μm; **f** Image analysis was performed to determine the percentage of GLS1-positive area per field under × 40 magnification based on six random fields per section (*n* = 24). For bar graph, one-way analysis of variance (ANOVA; mean ± SEM) was used to determine the *p-*value, ***p < 0.0001

We further evaluated if GLS1 is involved in regulating invasion and metastasis in the colorectal cancer and its association with clinical features. We performed immunohistochemistry (IHC) to analyze the expression of GLS1 and GLS2 in tissue microarrays, which contained colorectal tissues (15 cases of normal colorectal tissue, 17 cases of inflammatory hyperplasia, 9 cases of colorectal adenoma, and 39 cases of colorectal adenocarcinoma) spanning the stages of colorectal cancer transformation. GLS1 was predominantly expressed in colorectal tumor tissue as compared with normal tissue (Fig. 2a). In contrast, GLS2 was mainly expressed in normal tissue but not in cancer tissue (Fig. 2b). Interestingly, the expression of GLS2 was replaced by GLS1 gradually during colorectal cancer development in tissue microarrays (Table S1). GLS1 expression was low in normal colorectal tissues, and slightly increased in inflammatory hyperplasia (5.9%, Table S1). GLS1 expression was progressively increased in parallel with tumor progression, and was highest in colorectal adenocarcinoma (66.7%, *p* < 0.0001, Table S1). Conversely, the intensity of GLS2 expression was relatively high in normal colorectal (53.3%) and inflammatory hyperplasia tissues (64.7%), and was low in colorectal adenocarcinoma (*p* < 0.0001, Table S1). The colorectal adenoma samples expressed low levels of both GLS1 and GLS2 (Table S1).

To investigate the correlation of GLS1 expression with clinicopathological parameters of colorectal cancer, we used IHC to examine the expression of GLS1 in tissue microarrays containing samples from 85 cases of colorectal cancer. The patient characteristics are described in Table S2. The expression of GLS1 was not correlated with the patient’s age, gender, or tumor grade (Table S2). However, high GLS1 expression was significantly correlated with the presence of lymph node metastasis (*p* = 0.001). Moreover, the expression of GLS1 in lymph node metastases was much higher than in their primary tumor tissues (Fig. 2d–f, *p* < 0.0001, *n* = 24), whereas GLS1 was not observed in their adjacent normal colorectal tissue (Fig. 2c–f, *p* < 0.0001). Advanced clinical stage was also highly correlated with GLS1 expression (*p* < 0.0001) (Table S2). Taken together, these data indicate that GLS1 expression is significantly associated with lymph node metastasis and advanced clinical stage, suggesting that GLS1 may be involved in regulating invasion and metastasis in colorectal cancer.

### Hypoxia induces HIF-1-dependent expression of *GLS1*

Intratumoral hypoxia has been demonstrated in many types of human cancer including colorectal cancer, and is associated with patient mortality. The transcription of many genes involved in invasion, metastasis, and metabolic reprogramming of cancer cells is activated in response to hypoxia in a HIF-dependent manner^10^. Gene expression data from 433 human colorectal cancer specimens in the TCGA database were used to compare levels of GLS1 mRNA with a HIF metagene signature based on the expression of VEGFA, PDGFB, LOX, CXCR3, ANGPTL4, L1CAM, SLC2A1, P4HA1, P4HA2, and MET mRNA, which are all HIF-regulated genes. Statistical analysis of individual genes in the HIF signature revealed that *GLS1* expression was significantly correlated with 9 out of 10 HIF target genes (Table S3). The *RPL4* gene was analyzed as a representative non-HIF target gene. In contrast, none of the HIF target genes were significantly correlated with *RPL4*. These data suggest that GLS1 mRNA expression may be HIF-regulated in human colorectal cancer.

**Fig. 3:**
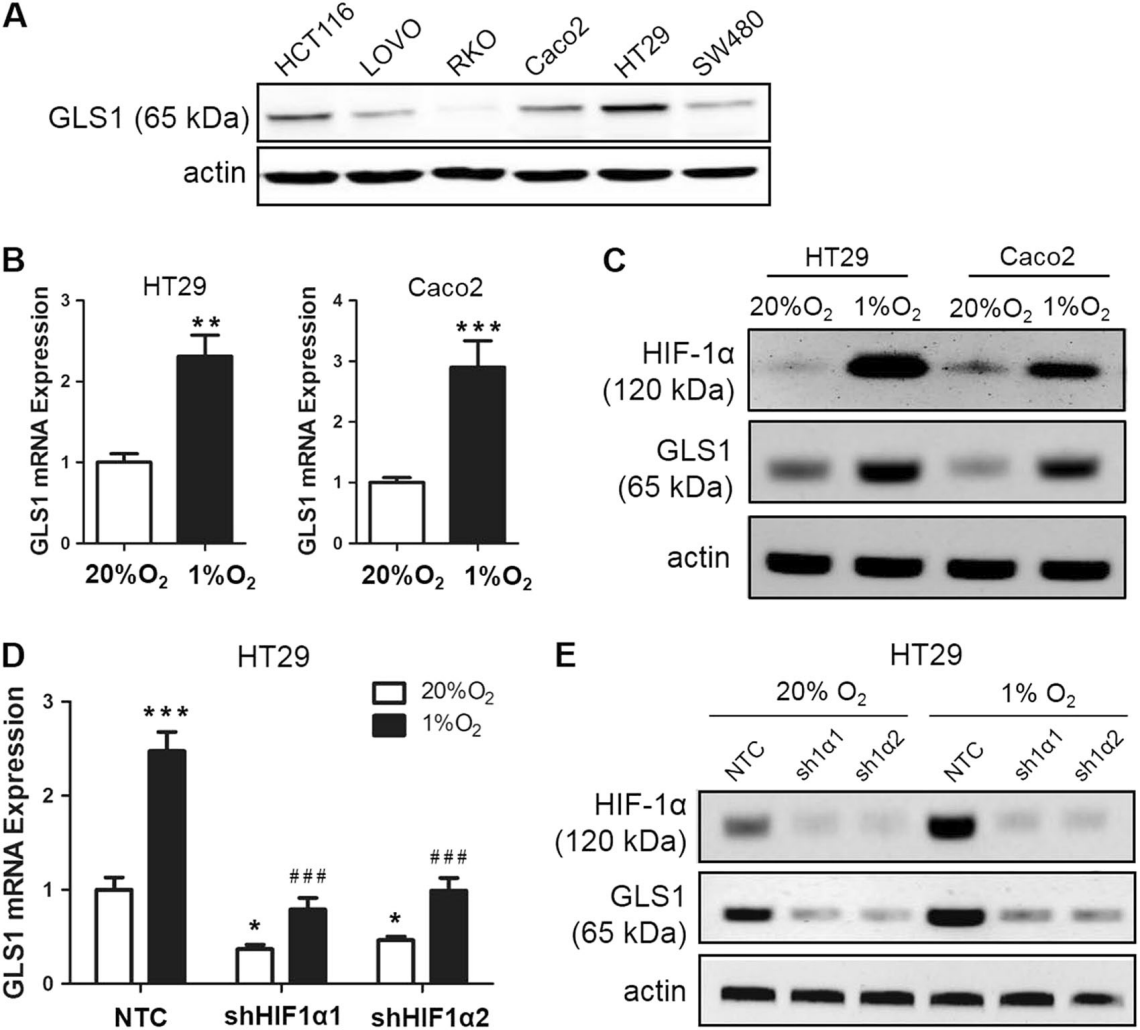
Hypoxia induces glutaminase 1 (GLS1) expression in a HIF-1α-dependent manner in colorectal cancer cell lines. **a** GLS1 protein expression in colorectal cancer cell lines. **b** Reverse transcription and quantitative real-time PCR (RT-qPCR) was performed to quantify GLS1 mRNA levels in colorectal cancer cell lines (HT29 and Caco2) following exposure to 20 or 1% O_2_ for 24 h. For each sample, the expression of GLS1 mRNA was quantified relative to 18S rRNA and then normalized to the result obtained from cells at 20% O_2_. Statistical analysis was performed before normalization. Data are shown as mean ± SEM; *n* = 3. **p < 0.01, ***p < 0.001 vs. 20% O_2_ (Student’s *t*-test). **c** Immunoblot assays were performed to analyze GLS1 and HIF-1α protein expression in HT29 and Caco2 cell lines following exposure to 20 or 1% O_2_ for 48 h. **d** levels of GLS1 mRNA were analyzed by RT-qPCR in HT29 subclones, which were stably transfected with non-targeting control (NTC) or vector encoding hypoxia-inducible factor (HIF)-1α short hairpin RNA (shRNA) (sh1α-1 or sh1α-2), and exposed to 20 or 1% O_2_ for 24 h (mean ± SEM; *n* = 3). *p < 0.05, ***p < 0.001 vs. NTC at 20% O_2_; ^###^*p* < 0.001 vs. NTC at 1% O_2_ (analysis of variance (ANOVA) with Bonferroni post-test). **e** Immunoblot assays were conducted using lysates prepared from HT29 subclones exposed to 20 or 1% O_2_ for 48 h

**Fig. 4:**
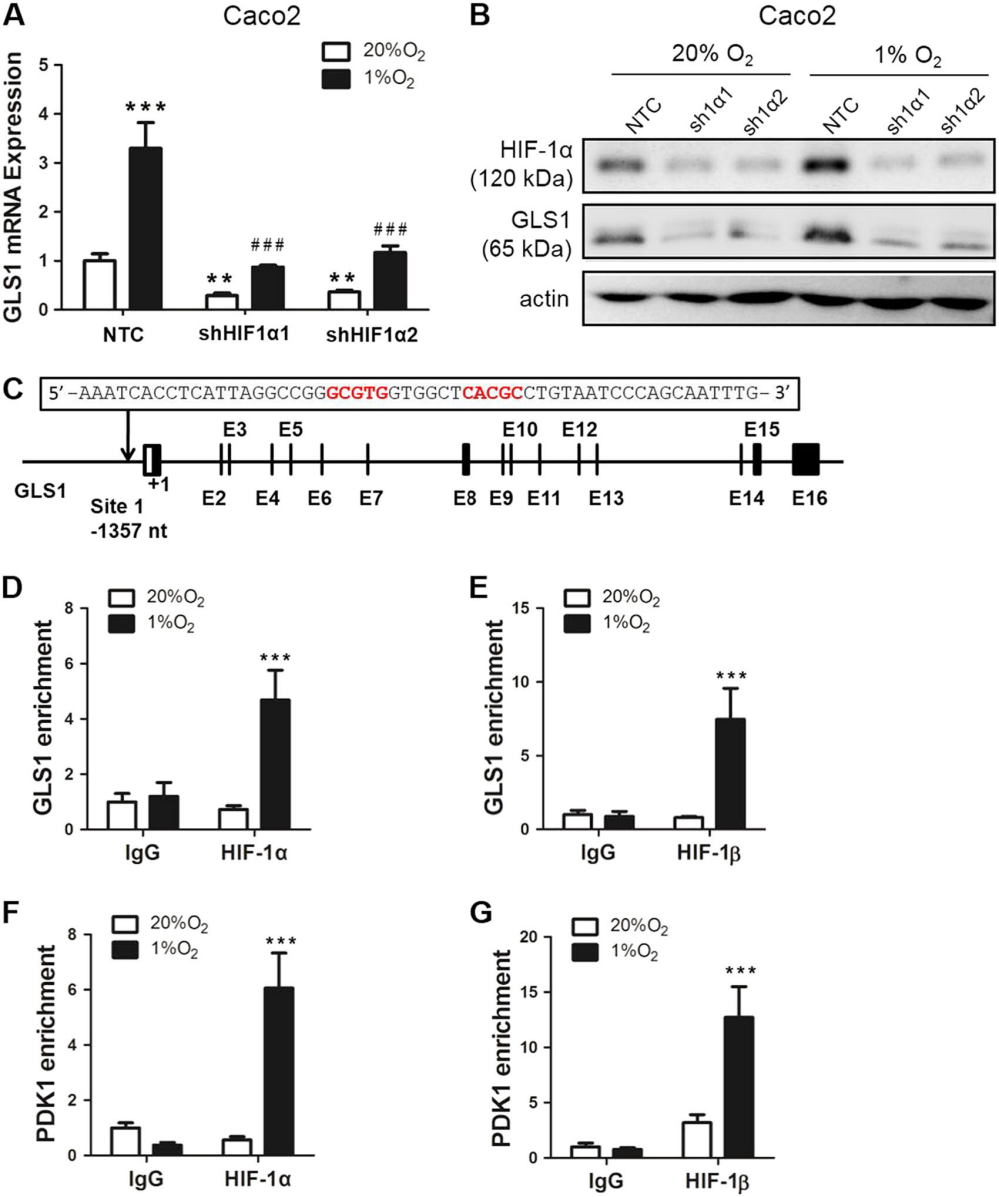
Hypoxia-inducible factor (HIF)-1α binds directly to the glutaminase 1 (GLS1) gene in hypoxic cells. **a** levels of GLS1 mRNA were analyzed by reverse transcription and quantitative real-time PCR (RT-qPCR) in Caco2 subclones (non-targeting control (NTC), sh1α-1, and sh1α-2), which exposed to 20 or 1% O_2_ for 24 h (mean ± SEM; *n* = 3). **p < 0.01, ***p < 0.001 vs. NTC at 20% O_2_; ^###^*p* < 0.001 vs. NTC at 1% O_2_ (ANOVA with Bonferroni post-test). **b** Immunoblot assays were conducted using lysates prepared from Caco2 subclones exposed to 20 or 1% O_2_ for 48 h. **c** HIF-binding sites in the 5′-flanking region of the human GLS1 gene were identified by ChIP as described below. *GLS* exons and hypoxia response element (HRE) are indicated by black bars and arrow. HRE nucleotide sequence is shown. **d**, **e** HT29 cells were exposed to 20 or 1% O_2_ for 16 h and ChIP assays were performed using IgG or antibodies against HIF-1α (**d**) and HIF-1β (**e**). Primers flanking the HRE were used for qPCR and results were normalized to lane 1 (mean ± SEM; *n* = 3). ***p < 0.001 vs. 20% O_2_ (analysis of variance (ANOVA) with Bonferroni post-test). **f**–**g** ChIP assays (HIF-1α and HIF-1β) for a known HIF-binding site in the *PDK1* gene are shown as positive controls. ***p < 0.001 vs. 20% O_2_ (ANOVA with Bonferroni post-test)

We next measured GLS1 protein levels in multiple colorectal cancer cell lines (HCT116, LOVO, RKO, Caco2, HT29, and SW480). GLS1 was highly expressed in highly metastatic cell lines (HCT116 and HT29), whereas GLS1 expression levels were relatively low in poorly and non-metastatic cell lines (LOVO, SW480, RKO, and Caco2) (Fig. 3a). GLS2 protein was not detected in most of the colorectal cancer cell lines (Fig. S2A), whereas it was slightly expressed in RKO cells, which had extremely low expression of GLS1 (Fig. S2A). As positive controls, GLS2 was highly expressed in a breast cancer cell line (MCF-7) (Fig. S2A) and a cervical cancer cell line (HeLa) (Fig. S2A), which confirmed results from our previous study^7^. To determine whether GLS1 expression is induced by hypoxia, GLS1 mRNA and protein levels were analyzed in HT29 and Caco2 cells, which were exposed to 20 or 1% O_2_ for 24 or 48 h. Reverse transcription (RT) and quantitative real-time PCR (qPCR) assays revealed that the expression of GLS1 mRNA (Fig. 3b) and protein (Fig. 3c) were greatly increased in both HT29 and Caco2 cells under hypoxic (1% O_2_) compared with non-hypoxic (20% O_2_) culture conditions. However, GLS2 mRNA was not changed in either HT29 or Caco2 cells under hypoxic compared with non-hypoxic conditions (Fig. S2B and S2C). To determine whether HIF-1α was required for GLS1 expression under hypoxic conditions, we analyzed HT29 and Caco2 subclones, which were stably transfected with an expression vector encoding short hairpin RNA (shRNA) targeting HIF-1α (sh1α-1 and sh1α-2) or a non-targeting control shRNA (NTC). Hypoxic induction of GLS1 mRNA expression was significantly decreased in HT29 (Fig. 3d) and Caco2 cells (Fig. 4a) when HIF-1α was silenced. Hypoxic induction of GLS1 protein expression was also abrogated in HT29 (Fig. 3e) and Caco2 cells (Fig. 4b) by two different shRNAs (sh1α-1 and sh1α-2) targeting HIF-1α. However, GLS1 expression was not regulated by HIF-2α (Fig. S2D–G). Taken together, these results indicate that GLS1 expression is induced by hypoxia in a HIF-1α-dependent manner.

### GLS1 is a direct HIF-1 target gene

HREs containing a match to the HIF-binding site consensus 5′-RCGTG-3′ have been identified in many HIF-1 target genes^31^. To determine whether *GLS1* was a direct HIF target gene, we analyzed the *GLS1* locus for the presence of matches to the consensus HIF-binding site sequence (5′-RCGTG-3′) located in DNase I-hypersensitive chromatin domains using the UCSC Genome Bioinformatics database (Fig. 4c). We identified four candidate sites that met these criteria (Fig. S3A), which were interrogated by chromatin immunoprecipitation (ChIP) assays of HT29 and Caco2 cells exposed to 20 or 1% O_2_ for 16 h (Fig. 4d, e and Fig. S3B, C). Antibodies against HIF-1α or HIF-1β and rabbit Ig (IgG) were used for ChIP. In HT29 cells, a single bona fide HIF-1-binding site was identified in the *GLS1* gene approximately 1.3 kb 5′ to the transcription start site (Fig. 4c), which showed significant hypoxia-induced binding of HIF-1α (Fig. 4d) and HIF-1β (Fig. 4e). ChIP assays of *PDK1*, which is a known HIF target gene, were also performed and revealed comparable hypoxia-induced binding of HIF-1α (Fig. 4f) and HIF-1β (Fig. 4g). We further confirmed our results by performing ChIP assays in Caco2 cells (Fig. S3B and S3C). Taken together, the data presented in Fig. 4 and Fig. S3 demonstrate that HIF-1, but not HIF-2 (Fig. S3D and S3E), bind directly to a site in the 5′-flanking region of the *GLS1* gene in hypoxic colorectal cancer cells.

### GLS1 deficiency impairs colorectal cancer cell invasion

**Fig. 5:**
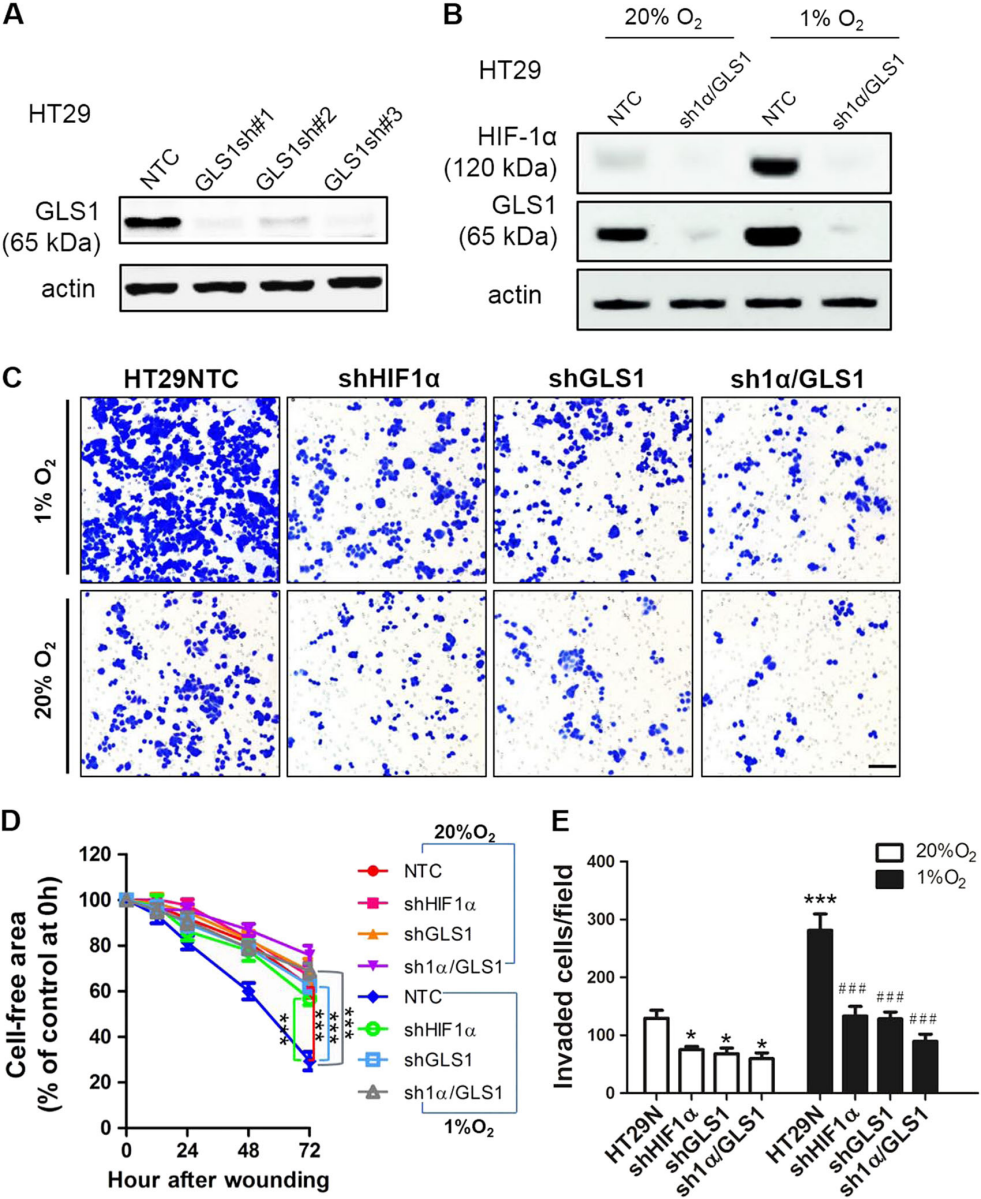
Glutaminase 1 (GLS1) deficiency suppresses colorectal cancer cell migration of invasion. **a** The protein levels of GLS1 in HT29 subclones transfected with lentiviral vectors encoding shRNA targeting GLS1 (shGLS1-1, shGLS1-2, shGLS1-3) and non-targeting control (NTC). **b** The protein levels of hypoxia-inducible factor (HIF)-1α and GLS1 in HT29 transfected with lentiviral vectors encoding short hairpin RNA (shRNA) targeting both HIF-1α and GLS1 (sh1α/GLS1) that exposed at 20 and 1% O_2_ for 48 h. **c** A total of 5 × 10^5^ cells were seeded on top of Matrigel-coated chamber inserts and incubated in serum-free Dulbecco’s modified Eagle’s medium (DMEM) at either 20 or 1% O_2_ for 48 h. The number of cells that invaded through the Matrigel to the underside of the filter was determined by staining with crystal violet and counting under bright field microscopy in 10 randomly selected fields. Scale bar = 100 μm. Each group was performed in triplicate, and all the results were repeated by three independent experiments. **d** Scratch assay was performed to analyze the migration of HT29 subclones (NTC, shHIF-1α, shGLS1, and sh1α/GLS1) by exposure to 20 or 1% O_2_ for 72 h. Percentage of cell-free area at indicated time points (0 h, 12 h, 24 h, 48 h, and 72 h) compared with that at 0 h was determined. Each condition was performed in triplicate. All data were repeated by three independent experiments and expressed as mean ± SEM (*n* = 3, ***p < 0.001). **e** The number of invaded cells per field was determined from 10 fields per filter. Mean ± SEM (*n* = 3) are shown. *p < 0.05, ***p < 0.001 vs. NTC at 20% O_2_; ^###^*p* < 0.001 vs. NTC at 1% O_2_

We further investigated whether GLS1 was required for colorectal cancer cell migration and invasion in vitro. HT29 and Caco2 cells were stably transfected with expression vectors encoding shRNA targeting GLS1 (shGLS1), or shRNAs targeting both HIF-1α and GLS1 (shHIF-1α/GLS1). The efficiency of protein knockdown was demonstrated by immunoblot assays: GLS1 knockdown (Fig. 5a and Fig. S4A), and HIF-1α/GLS1 double knockdown (Fig. 5b and Fig. S4B). We then measured the migration of the HT29 subclones (NTC, shHIF-1α, shGLS1, and shHIF-1α/GLS1) in a scratch assay after exposure to 20 or 1% O_2_ for 72 h. The remaining cell-free area at each time point (0 h, 12 h, 24 h, 48 h, or 72 h) as a percentage of the initial area was taken as an index of cell migration (Fig. 5d, S5A, B). Hypoxia significantly increased the migration of NTC cells into the cleared area; however, knockdown of GLS1, HIF-1α, or HIF-1α/GLS1 blocked the hypoxia-induced migration (Fig. 5d, S5A, B). Under non-hypoxic conditions, knockdown of GLS1, HIF-1α, or HIF-1α/GLS1 slightly suppressed migration compared with NTC knockdown, but the suppression was not significant within 72 h (Fig. 5d, S5A, B).

Next, HT29 subclones were incubated at 20 or 1% O_2_ for 48 h, and then assayed for their ability to invade through Matrigel. Compared with NTC cells incubated at 20% O_2_, NTC cells incubated at 1% O_2_ showed increased invasion through Matrigel (Fig. 5c–e). Hypoxia-induced invasion of HIF-1α or GLS1 knockdown cells was significantly decreased, particularly in HIF-1α/GLS1 double knockdown cells. Invasion of HIF-1α, GLS1, or HIF-1α/GLS1 knockdown cells was also slightly impaired under normoxic condition (Fig. 5c–e). Similar results were obtained when we performed the scratch assay (Fig. S4D and S6) and transwell invasion assay (Fig. S4C and S4E) using Caco2 subclones (NTC, shHIF-1α, shGLS1, and shHIF-1α/GLS1). These data indicate that GLS1 is required for the migration and invasion of colorectal carcinoma cells, particularly under hypoxic conditions.

We have also studied whether hypoxia alters GLS1 activity by measuring glutamine consumption and glutamate production. Data in Figure S7 showed that hypoxia significantly enhances glutaminolysis, as indicated by increased glutamine consumption (Fig. S7A and S7B) and glutamate concentration (Fig. S7C and S7D) in the culture medium of HT29 or Caco2 subclones (NTC, shHIF-1α, and shGLS1), compared with non-hypoxic culture conditions. Knockdown of GLS1 led to significantly decreased glutamine consumption (Fig. S7A and S7B) and glutamate concentration (Fig. S7C and S7D) in both hypoxic and non-hypoxic culture conditions. Knockdown of HIF-1α also significantly decreased glutamine consumption (Fig. S7A and S7B), and to a greater extent the glutamate concentration (Fig. S7C and S7D), particularly under hypoxic culture conditions.

### GLS1 deficiency suppressed hypoxia-induced lung colonization and lymph node metastasis of colorectal cancer in vivo

**Fig. 6:**
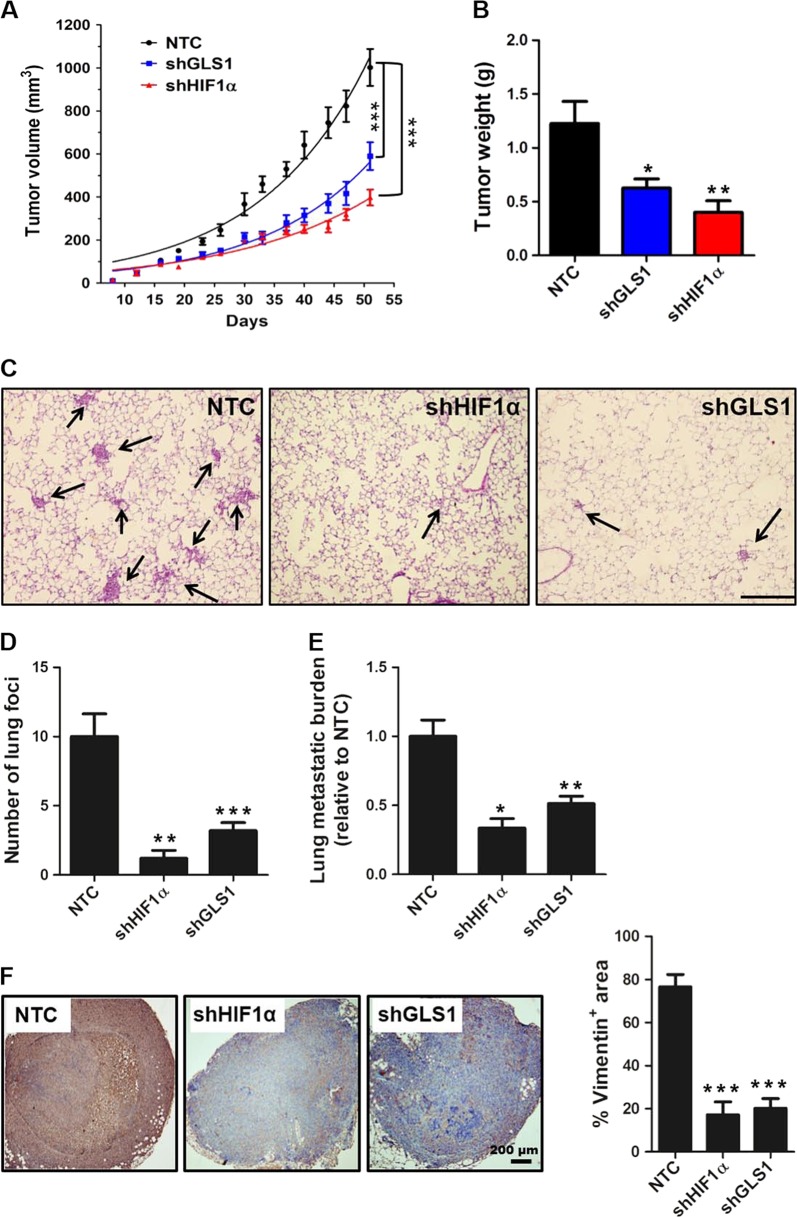
Glutaminase 1 (GLS1) deficiency suppresses colorectal tumor growth and metastatic colonization. **a** HT29 subclones (2 × 10^6^ cells) were implanted into the groin of 6–8-week-old male SCID mice. Primary tumor volume was determined twice per week. After 51 days, the primary tumor was harvested and weighed. ***p < 0.001 vs. non-targeting control (NTC) by two-way analysis of variance (ANOVA) with Bonferroni post-test (mean ± SEM; *n* = 6). **b** Tumor weights are shown. **c** HT29 subclones (1 × 10^6^ cells) were injected into the tail vein of SCID mice. Four weeks later, the left lung was fixed under inflation and sections were stained with hematoxylin and eosin to identify metastatic foci (arrows). Scale bar = 200 μm. **d** The number of metastatic foci per field (six random fields per section) was determined. **e** DNA was extracted from the right lung and analyzed by qPCR using primers specific for human *HK2* gene sequences to quantify the total lung metastatic burden. **f** The inguinal lymph node ipsilateral to the hind paw pad injection was harvested and analyzed by immunohistochemistry using an antibody specific for human vimentin (left panel). Image analysis was performed to determine the vimentin positive area per field under × 40 magnification based on six random fields per section (right panel). For all bar graphs, *p < 0.05, **p < 0.01, ***p < 0.001 vs. NTC by ANOVA with Bonferroni post-test (mean ± SEM)

To determine whether GLS1 affects the proliferation of colorectal cancer cells in vivo, we injected 2 × 10^6^ of HT29 cells (NTC, shHIF-1α, or shGLS1 subclone) subcutaneously into the inguinal region of SCID mice (*N* = 6 mice in each group). Tumor growth was monitored twice per week. After 51 days, tumors were excised and weighed. Compared with tumors from the NTC group, tumors formed by shGLS1 or shHIF-1α cells were significantly decreased in their growth rate (Fig. 6a) and final weight (Fig. 6b). Consistent with the reduction in tumor growth, Ki67 staining (Fig. S8A) was significantly decreased in tumors formed by shGLS1 or shHIF-1α cells (Fig. S8B).

Next, HT29 cells (NTC, shHIF-1α, or shGLS1 subclone) were incubated under hypoxic conditions for 48 h, and then injected into the tail vein of nude mice (*N* = 6 mice in each group). Four weeks later, the lungs were harvested for hematoxylin and eosin staining (Fig. 6c) and the number of metastatic foci was determined (Fig. 6d). In addition, the total colorectal cancer cell burden was determined by qPCR using human-specific *HK2* primers (Fig. 6e). Compared with the NTC group, lung colonization was markedly reduced in GLS1 and HIF-1α knockdown groups.

Finally, HT29 subclones (NTC, shHIF-1α, and shGLS1) were injected subcutaneously into the left hind paw pad of SCID mice (*N* = 6 per group). After 6 weeks, the mice were euthanized and inguinal lymph nodes were harvested for human vimentin IHC as a measure of lymph node colonization by cancer cells. Compared with the NTC group, human vimentin staining was significantly decreased in ipsilateral inguinal lymph nodes harvested from the HIF-1α or GLS1 knockdown group (Fig. 6f). Taken together, the data indicate that GLS1 is required for colorectal tumor growth and metastatic colonization.

## Discussion

Intratumoral hypoxia increases the accumulation of HIF-1α, which regulates multiple downstream target genes associated with cancer migration, invasion, metastasis, and relapse^10^. In the process of cancer metastasis, HIF has been shown to increase vascular permeability and promote cancer cell intravasation by inducing vascular endothelial growth factor (VEGF) activity;^24^ HIF-dependent expression of L1 cell adhesion molecule (L1CAM)^32^, and angiopoietin-like 4 (ANGPTL4)^33^ mediate breast cancer cell adherence to endothelial cells (ECs) and disruption of tight junctions between ECs, respectively; and lysyl oxidase (LOX), an extracellular enzyme that is upregulated by HIF-1, leads to collagen crosslinking and the recruitment of bone marrow-derived cells that together define the pre-metastatic niche^34^. Recently, HIF-dependent expression of the serine synthesis pathway enzyme phosphoglycerate dehydrogenase was shown to be required for breast cancer metastasis^29^.

In this study, we have demonstrated that HIF-1 stimulates the expression of GLS1, a key enzyme in glutamine metabolism, to promote colorectal cancer cell migration, invasion, and metastasis. Analysis of TCGA colorectal cancer data revealed that GLS1 expression is significantly correlated with a HIF metagene signature, which is based on the combined expression of 10 HIF-1 target genes implicated in breast cancer metastasis (*VEGFA*, *PDGFB*, *LOX*, *CXCR3*, *ANGPTL4*, *L1CAM*, *SLC2A1*, *P4HA1*, *P4HA2*, and *MET*). GLS1 mRNA and protein levels are increased when colorectal cancer cell lines are incubated under hypoxic conditions. HIF-1 binds to an HRE in the *GLS1* gene, leading to increased GLS1 mRNA and protein expression. By performing a series of experiments in vitro and in vivo, we found that GLS1 is required for colorectal cancer cell migration, invasion, primary tumor growth, and metastatic colonization of lymph nodes and lungs. Thus, the HIF-1-mediated increase in GLS1 expression provides a novel molecular mechanism by which intratumoral hypoxia promotes the invasion and metastasis of colorectal cancer.

Cancer cells exhibit upregulated aerobic glycolysis, glutaminolysis, and fatty acid synthesis for proliferation. In addition to the established function of metabolic enzymes, recent studies have focused on novel roles in maintaining malignant phenotypes. Both HIF-1 and c-Myc mediate transcriptional upregulation of multiple glycolytic enzymes^35^. HIF-1 has also been shown to play an important role in regulating proteins in glutaminolysis pathway and fatty acid synthesis, such as glutamate transporters (SLC1A1, SLC1A3)^36^, glutamate receptors (GRIA2, GRIA3)^36^, lipin 1 (LPIN1)^37^, and fatty acid synthase (FASN)^38^. In the present study, our data demonstrate for the first time that HIF-1 stimulates glutamine metabolism by elevating GLS1 expression and activity in colorectal cancer. Our results show that GLS1 is overexpressed in several human cancers including colorectal, esophageal, gastric, hepatocellular, and head and neck squamous cell cancer, as compared with their adjacent normal tissues. Increased *GLS1* expression in a variety of human cancer types was associated with significantly decreased patient survival, which suggests that GLS1 may function as a potential prognostic biomarker for many human cancers. Additionally, we have demonstrated a positive correlation between GLS1 protein expression and clinicopathological features of colorectal cancer, such as the presence of lymph node metastasis and advanced clinical stage, which is consistent with our finding that GLS1 promotes progression and metastasis of colorectal cancer cells in vitro and in vivo. Intriguingly, we observed increased GLS1 expression in lymph node metastases of colorectal cancer patients as compared with their primary tumor. Similarly, HIF-1α protein levels are also increased in metastases of cancer patients^39^, suggesting a potential regulatory link between HIF-1α and GLS1. Regarding the potential role of GLS1 in promoting metastasis, one group of researchers proposed that GLS1 may initiate metastasis though induction of the epithelial–mesenchymal transition^40^. But based on our previous studies, we found that induction of glutaminase increased the level of glutathione and NADPH, and decreased the production of ROS in cervical cancer cells^7^. Glutathione is the major antioxidant in cells and is synthesized from glutamate, cysteine, and glycine^41^. We also found that hypoxia enhances glutaminolysis via increased *GLS1* transcription, which could further promote glutathione synthesis. It has been reported recently that glutathione plays an important role in regulating the expression of Nanog and other pluripotency factors that determine the breast cancer stem cell (CSC) phenotype^42^. CSCs are a small subpopulation of cells with the capability for self-renewal that have been implicated in cancer recurrence, proliferation, and metastasis^43,44^. Our data indicate that in the hypoxic tumor microenvironment, GLS1 high expression promotes colorectal carcinoma metastasis and it is possible that this might involve induction of CSC phenotype. Further studies will be needed to determine whether this mechanism contributes to the observed effects of GLS1 knockdown on colorectal cancer cell migration, invasion, and metastatic colonization.

## Materials and methods

### Statistical analysis of microarray data

Gene expression data from the Colon & Rectum Adenocarcinoma dataset of TCGA were obtained from https://genome-cancer.ucsc.edu. Pearson’s correlation coefficient was used to determine *p*-values for co-expression. For *GLS1* gene expression analysis, data were obtained from the TCGA or Oncomine database (www.oncomine.org). Mann–Whitney *U-*test or analysis of variance (ANOVA) followed by Bonferroni post-test for multiple comparisons was used to determine *p*-values for the comparison of gene expression levels.

### Survival analysis

Prognostic significance of GLS1 mRNA expression in different types of cancer was analyzed using Kaplan-Meier (KM) plotter (www.kmplot.com)^45^, NCBI Gene Expression Omnibus http://www.ncbi.nlm.nih.gov/geo datasets GSE17538, GSE38832, GSE39084, and GSE39582 and GEPIA database (http://gepia.cancer-pku.cn)^46^. Briefly, GLS1 was entered into the online database to obtain Kaplan–Meier survival plots in which the number at risk is indicated below the main plot. The log-rank *p-*values were calculated and displayed on the webpage. The survival rates were investigated with median cut-off (low represents patients with GLS1 mRNA levels less than the median; high represents patients with GLS1 mRNA levels greater than the median).

### Clinical and specimens

All slides were prepared from stored pretreatment paraffin-embedded tissue blocks from colorectal cancer patients who underwent surgery at Southwest Hospital. All specimens were confirmed by pathological examination, and staging was performed according to the 1997 CRC staging system of the UICC. Briefly, 165 colorectal cancer specimens were made into two tissue microarrays using the tissuearrayer TMA-1 (Beecher Instruments)^47^. Clinical investigation complied with the ethical standards codified in the 1964 Declaration of Helsinki. The protocol of IHC for patient tissues was approved by the Ethics Committee of the First Affiliated Hospital (Southwest Hospital), Third Military Medical University (Permit Number: 2012[12]), and all patients or family members involved provided written informed consent.

### Cell culture

All colorectal cancer cell lines were obtained from the ATCC (Manassas, VA, USA) and cultured in Dulbecco’s modified Eagle’s medium (GIBCO) or RPMI-1640 medium (GIBCO) supplemented with 10% fetal bovine serum (FBS; GIBCO), penicillin (100 U/mL), and streptomycin (0.1 mg/mL) (Beyotime Institute of Biotechnology, China). Cells were incubated at 37 °C in a humidified atmosphere containing 5% CO_2_. For hypoxic exposure, cells were placed in a modular incubator chamber that was flushed with a 1% O_2_/5% CO_2_/94% N_2_ gas mixture and sealed.

### ShRNA, lentiviruses, and transduction

The pLKO.1-puro lentiviral vectors encoding shRNA targeting HIF-1α (sh1α-1, clone ID: NM_001530.x-2671s1c1; sh1α2, clone ID: NM_001530.x-1048s1c1), HIF-2α (sh2α-1, clone ID: NM_001430.x-1694s1c1; sh2α-2, clone ID: NM_001430.x-2419s1c1) and NTC were purchased from Sigma. The pLKO.1-puro lentiviral vectors encoding shRNA targeting GLS1 were purchased from Sigma-Aldrich: shGLS1-1 (clone ID: NM_014905.2-1441s1c1); shGLS1-2 (clone ID: NM_014905.2-2213s1c1); shGLS1-3 (clone ID: NM_014905.2-1576s1c1). Lentiviral vectors were co-transfected with plasmid pCMV-dR8.91 and a plasmid encoding vesicular stomatitis virus G protein into 293T cells using Lipofectamine 2000 (Invitrogen). Medium containing viral particles was collected 48 h after transfection and passed through a 0.45-μM filter. HT29 and Caco2 cells were transduced with viral supernatant supplemented with 8 μg/mL Polybrene (Sigma-Aldrich). After 24 h, cells were selected in medium containing 0.6 μg/mL puromycin (Sigma-Aldrich).

### RT-qPCR

Total cellular RNA was extracted using TRIzol (Invitrogen), precipitated with isopropanol, treated with DNase I (Ambion), and reverse transcribed with the iScript cDNA Synthesis kit (Bio-Rad). qPCR analysis was performed using SYBR Green (Bio-Rad) with the iCycler Real-time PCR Detection System (Bio-Rad). The 2^−ΔΔCt^ method was used to calculate the relative gene expression^32^. Results were normalized to 18S rRNA expression. Primer sequences are listed in Table S4.

### Immunoblot assay

Immunoblot assays were performed as previously described^17^. Aliquots of whole-cell lysates prepared in RIPA lysis buffer were fractionated by 8 or 10% SDS/PAGE (sodium dodecyl sulfate/polyacrylamide gel electrophoresis). Antibodies used in immunoblot assays were: HIF-1α (BD Transduction Laboratory); HIF-2α (Cell signaling); GLS1 (Epitomics); GLS2 (Abcam); β-actin (Santa Cruz). HRP (horseradish peroxidase)-conjugated anti-rabbit and anti-mouse secondary antibodies (Santa Cruz) were used. Chemiluminescent signal was developed using ECL Plus (GE Healthcare).

### ChIP assay

ChIP assays were performed as previously described^17^. HT29 and Caco2 cells were cross-linked in 3.7% formaldehyde for 10 min and lysed with SDS lysis buffer. Chromatin was sheared by sonication and lysates were pre-cleared with salmon sperm DNA/protein A-agarose slurry (Millipore) and incubated with IgG (Novus Biologicals) or antibodies against the following proteins: HIF-1α (Santa Cruz), HIF-2α (Cell signaling), HIF-1β (Novus Biologicals), and GLS1 (Epitomics). Salmon sperm DNA/protein A-agarose slurry was added and the agarose beads were washed sequentially with: low- and high-salt immune complex wash buffers; LiCl immune complex wash buffer; and twice with TE (Tris-EDTA) buffer (10 mM Tris-HCl/1 mM EDTA). DNA was eluted in 1% SDS with 0.1 M NaHCO_3_, and crosslinks were reversed by addition of 0.2 M NaCl. DNA was purified by phenol–chloroform extraction and ethanol precipitation, suspended in 50 μl TE buffer, and a 2-μl aliquot was used for qPCR. Primer sequences are listed in Table S4.

### Extraction of genomic DNA

Lung tissue was digested with proteinase K at 55 °C overnight, and genomic DNA was extracted with phenol and chloroform, and precipitated with isopropanol. In all, 200 ng of genomic DNA was used for qPCR using human *HK2* gene-specific primers to quantify lung metastatic burden. The result was normalized to the result obtained using primers that bind to both human and mouse 18S rRNA genes.

### IHC

Xenografts or lymph nodes were fixed in 10% formalin and paraffin embedded. Sections and tissue microarrays were dewaxed in xylene, hydrated with graded ethanol, followed by antigen retrieval using citrate buffer (pH 6.1). The instantaneous SP supersensitive kit (SP-9000, Beijing Zhongshan Jinqiao Biotechnology Co., Ltd) was used with antibodies against GLS1 (Epitomics), GLS2 (Epitomics), Ki67 (Abcam), and vimentin (Santa Cruz). Sections were counterstained with Mayer’s hematoxylin (Sigma).

### Criteria for assessing immunohistochemical results

For each sample, five random fields were selected for scoring and a mean score of each slide was calculated in final analysis. The percentage of stained cells was scored as follows: 0 (no positive cells), 1 (<10% positive cells), 2 (10–40% positive cells), 3 (40–70% positive cells), and 4 (>70% positive cells). The intensity of positive staining was scored as follows: 0 (negative staining), 1 (weak staining exhibited as light yellow), 2 (moderate staining exhibited as yellow brown), and 3 (strong staining exhibited as brown). The proportion and intensity scores are added to obtain a total score (TS range: 0, 2–7). Samples were divided into two categories depending on the IHC score: category low corresponded to IHC score <4; Category high corresponded to IHC score ≥4. Slides were examined and scored independently using GOG criteria^48^ by two histopathologists (S.L. Xu and J Yang) blinded to other pathological information. For tumor growth and lymph nodes metastasis analysis, immunohistochemical staining of Ki67 and vimentin was quantified by Image J software (NIH) as described previously^49^. Ki67 staining were calculated as the number of DAB (3,3'-diaminobenzidine tetrahydrochloride)-stained cells divided by the number of total cells per field. The extent of vimentin staining was calculated as the DAB-positive area divided by the hematoxylin-positive area. For quantitative measurement of GLS1-positive staining on clinical samples (adjacent normal colorectal tissue, primary colorectal tumor and lymph node metastasis), immunohistochemical staining of GLS1 was quantified by Image J software (NIH) as described above. The percentage of GLS1-positive area was calculated as the DAB-positive area divided by the hematoxylin-positive area.

### Cell migration (scratch) assay

Cells were seeded in 24-well plates at 1 × 10^6^ cells/well, grown to confluence and a 1-mL pipette tip was dragged through the monolayer. Cells were washed to remove cellular debris and allowed to migrate for 72 h under hypoxic or normoxic conditions. Images were taken at 0 h, 12 h, 24 h, 48 h, and 72 h after scratching under a Leica phase-contrast microscope. The relative surface area traveled by the leading edge was assessed by using NIH Image J image analysis software. Cell migration effect was calculated as the percentage of the remaining cell-free area compared with the initial cell-free area. All experiments were performed in triplicate and repeated three times.

### Matrigel invasion assay

All cells were cultured in serum-free medium at 20 or 1% O_2_ for 24 h. A total of 5 × 10^5^ cells were seeded into the upper well of a 24-well Transwell chamber insert (Fisher Scientific) that was coated with Matrigel (BD Biosciences) and incubated in serum-free medium. Lower wells of the transwells contained the same medium supplemented with 10% FBS. Invasion of the cells through Matrigel to the underside of the filter was assessed after 48 h under hypoxic or normoxic conditions by staining with crystal violet and counting 10 randomly selected fields under bright field microscopy.

### Glutamine and glutamate detection

Cells were cultured for 48 h in six-well plates in phenol red-free medium under hypoxic or non-hypoxic conditions. The culture medium was collected and cells were lysed with RIPA buffer. Concentrations of glutamine in the medium and in the cell lysate were determined with the glutamine/glutamate determination kit (GLN-1; Sigma-Aldrich). The dehydrogenation of glutamate to α-ketoglutarate in samples was accompanied by reduction of NAD^+^ to NADH, which was measured using a spectrophotometer at 340 nm, and was proportional to the amount of glutamate. A standard curve was determined for each experiment to calculate the concentration of glutamate in samples. Glutamine levels were determined from the amount of glutamine converted to glutamate via glutaminase. The glutamine consumption was calculated as the difference between the initial and final glutamine levels of the cells in culture and was normalized to total protein amounts. Glutamate production was calculated as the difference between the final and initial levels of glutamate and normalized to total protein amounts.

### Animal studies

Animal experiments were approved by the Institutional Animal Care and Use Committee of Sichuan University (Chengdu, China). Six to 8-week-old male SCID mice were purchased from the Laboratory Animal Centre of Sichuan University and the Institute of Experimental Animal of Third Military Medical University. HT29 (NTC, shHIF-1α, or shGLS1) cells were harvested by trypsinization, resuspended at 10^7^ cells/mL in a 50:50 mix of PBS (phosphate-buffered saline):Matrigel (BD Biosciences), and 2 × 10^6^ cells were injected subcutaneously into the inguinal area of mice (six mice per group). Tumor volume (mm^3^) was calculated as 0.52 × L × W × T (length, width, and thickness). Tumor volumes and body weights were monitored twice per week. After 51 days, tumors were excised and weighed. Xenografts were harvested to be subjected to IHC staining.

For the lung colonization assay, 1 × 10^6^ HT29 (NTC, shHIF-1α, or shGLS1) cells were incubated at 1% O_2_ for 48 h. Cells were injected into the tail vein of 6-week-old male SCID mice, respectively (*n* = 6 per group). After 4 weeks, the left lung was inflated with low melting point agarose for formalin fixation and paraffin embedding. Sections were stained with hematoxylin and eosin. The right lung was used for genomic DNA extraction.

For the lymphatic metastasis assay, 1 × 10^6^ HT29 (NTC, shHIF-1α, or shGLS1) cells in 0.04 mL PBS were injected subcutaneously into the left hind paw pad of SCID mice respectively (*n* = 6 per group). After 6 weeks, mice were sacrificed, and the inguinal lymph nodes were harvested to be subjected to IHC staining.

### Statistical analysis

All data were expressed as mean ± SEM. When data were not Gaussian, a logarithmic or square root transformation was applied. Differences between two experimental groups were assessed using Student’s test or two-way ANOVA followed by Bonferroni post-test. Multiple groups were analyzed by ANOVA followed by Bonferroni post-test. A *p-*value of < 0.05 was considered statistically significant.

## Supplementary information

supplemental figure legend

supplemental figures

supplemental tables
