# Supplementary material for: Glutaminase 1 expression in colorectal cancer cells is induced by hypoxia and required for tumor growth, invasion, and metastatic colonization
Source: Cell Death Dis. 2019 Jan 17;10(2):40. doi: 10.1038/s41419-018-1291-5 (PMC6426853; doi:10.1038/s41419-018-1291-5)
Supplement: Supplementary file 1 — supplemental figure legend [file 41419_2018_1291_MOESM1_ESM.docx]

**SUPPLEMENTAL FIGURE LEGEND**

**Figure S1.** Analysis of GLS1 mRNA expression levels in human cancer and normal tissue from Oncomine database. A, Light blue, samples from normal brain tissue (n=23); Dark blue, samples from glioblastoma (n=81), *P* = 6.33e-14. B, Light blue, samples from normal colorectal tissue (n=24); Dark blue, samples from colorectal carcinomas (n=36), *P* = 3.48e-11. C, Light blue, samples from normal breast tissue (n=3); Dark blue, samples from invasive ductal breast carcinoma (n=34), *P* = 5.11e-5.

**Figure S2.** A, GLS2 protein expression in colorectal cancer cell lines (HCT116, LOVO, HT29, SW480, RKO and Caco2). Breast cancer cell line (MCF-7) and cervical cancer cell line (Hela) are positive expression controls. B-C, RT-qPCR was performed to quantify GLS2 mRNA levels in colorectal cancer cell lines (HT29 and Caco2) following exposure to 20% or 1% O_2_ for 24 h. Data are shown as mean ± SEM; n = 3. ns = not significant. D-E, The protein levels of HIF-2α in HT29 (D) and Caco2 (E) subclones transfected with lentiviral vectors encoding shRNA targeting HIF-2α (shHIF2α-1 and shHIF2α-2) or non-targeting control (NTC) that exposed at 20% and 1% O_2_ for 48 h. F-G, levels of GLS1 mRNA were analyzed by RT-qPCR in HT29 (F) and Caco2 (G) subclones (NTC, shHIF2α-1 and shHIF2α-2) that exposed to 20% or 1% O_2_ for 24 h (mean ± SEM; n = 3). ****P* < 0.001 vs. NTC at 20% O_2_ (ANOVA with Bonferroni post-test).

**Figure S3.** A, candidate HIF binding sites in the GLS1 gene. Four sequences (1–4) matching the consensus 5′-RCGTG-3′ (R = A or G; shown in red) and located in a DNase I hypersensitive region of chromatin (coordinates given relative to the transcription start site) were tested for binding of HIF-1α and HIF-1β by chromatin immunoprecipitation assay. HIF-1 was shown to bind selectively to site 1 (see Fig. 4C-4E). H3K4Me1 and H3K4Me3, monomethylation and trimethylation, respectively, of lysine residue 4 of histone H3; H3K27Ac, acetylation of lysine-27 of histone H3. B-C, Caco2 cells were exposed to 20% or 1% O_2_ for 16 h and ChIP assays were performed using IgG or antibodies against HIF-1α (B) and HIF-1β (C). Primers flanking the HRE were used for qPCR and results were normalized to lane 1 (mean ± SEM; n = 3). **P* < 0.05, ****P* < 0.001 vs. 20% O_2_ (ANOVA with Bonferroni post-test). D-E, HT29 (D) or Caco2 (E) cells were exposed to 20% or 1% O_2_ for 16 h and ChIP assays were performed using IgG or antibodies against HIF-2α. Primers flanking the HRE were used for qPCR and results were normalized to lane 1 (mean ± SEM; n = 3).

**Figure S4.** GLS1 knockdown suppress colorectal cancer cell invasion. A, The protein levels of GLS1 in Caco2 subclones transfected with lentiviral vectors encoding shRNA targeting GLS1 (shGLS1-1, shGLS1-2, shGLS1-3) and non-targeting control (NTC). B, The protein levels of HIF-1α and GLS1 in Caco2 transfected with lentiviral vectors encoding shRNA targeting both HIF-1α and GLS1 (sh1α/GLS1) that exposed at 20% and 1% O_2_ for 48 h. C, A total of 5 × 10^5^ cells were seeded on top of Matrigel-coated chamber inserts and incubated in serum-free DMEM at either 20% or 1% O_2_ for 48 h. The number of cells that invaded through the Matrigel to the underside of the filter was determined by staining with crystal violet and counting under bright field microscopy in 10 randomly selected fields. Scale bar=100 μm. Each group was performed in triplicate, and all the results were repeated by three independent experiments. D, Scratch assay was performed to analyze the migration of Caco2 subclones (NTC, shHIF-1α, shGLS1 and sh1α/GLS1) by exposure to 20% or 1% O_2_ for 72 h. Percentage of cell-free area at indicated time points (0 h, 12 h, 24 h, 48 h and 72 h) compared with that at 0 h was determined. Each condition was performed in triplicate. All data were repeated by three independent experiments and expressed as mean ± SEM (n = 3, ****P* < 0.001). E, The number of invaded cells per field was determined from 10 fields per filter. Mean ± SEM (n = 3) are shown. ***P* < 0.01, ****P* < 0.001 vs. NTC at 20% O_2_; ^###^*P* < 0.001 vs. NTC at 1% O_2_.

**Figure S5.** Representative images of scratch assay of HT29 subclones (NTC, shHIF-1α, shGLS1 and sh1α/GLS1) are shown at 0 h and 72 h under 20% or 1% O_2_ conditions. Black lines represent the leading edges of the migration front.

**Figure S6.** Representative images of scratch assay of Caco2 subclones (NTC, shHIF-1α, shGLS1 and sh1α/GLS1) are shown at 0 h and 72 h under 20% or 1% O_2_ conditions. Black lines represent the leading edges of the migration front.

**Figure S7.** Determination of glutamine consumption and glutamate concentration. HT29 or Caco2 subclones (NTC, shHIF-1α and shGLS1) were cultured under 20% or 1% O_2_ conditions for 48 h, and then glutamine consumption and glutamate concentration in HT29 subclones (A and C) and Caco2 subclones (B and D) were measured. Mean ± SEM (n = 3) are shown. **P* < 0.05, ***P* < 0.01, ****P* < 0.001 vs. NTC at 20% O_2_; ^###^*P* < 0.001 vs. NTC at 1% O_2_.

**Figure S8.** Staining and quantification of Ki67 positive cells. A, immunohistochemistry was performed on tumor tissue sections from mice injected with HT29 subclones (NTC, shGLS1 or shHIF-1α) to analyze cell proliferation by Ki67 staining. Scale bar =200 μm. B, the stained sections were subjected to image analysis and quantification (mean ± SEM); ***p <0.001 vs NTC, one-way ANOVA.
