# Supplementary material for: Glutaminase 1 expression in colorectal cancer cells is induced by hypoxia and required for tumor growth, invasion, and metastatic colonization
Source: Cell Death Dis. 2019 Jan 17;10(2):40. doi: 10.1038/s41419-018-1291-5 (PMC6426853; doi:10.1038/s41419-018-1291-5)
Supplement: Supplementary file 2 — supplemental figures [file 41419_2018_1291_MOESM2_ESM.pdf]

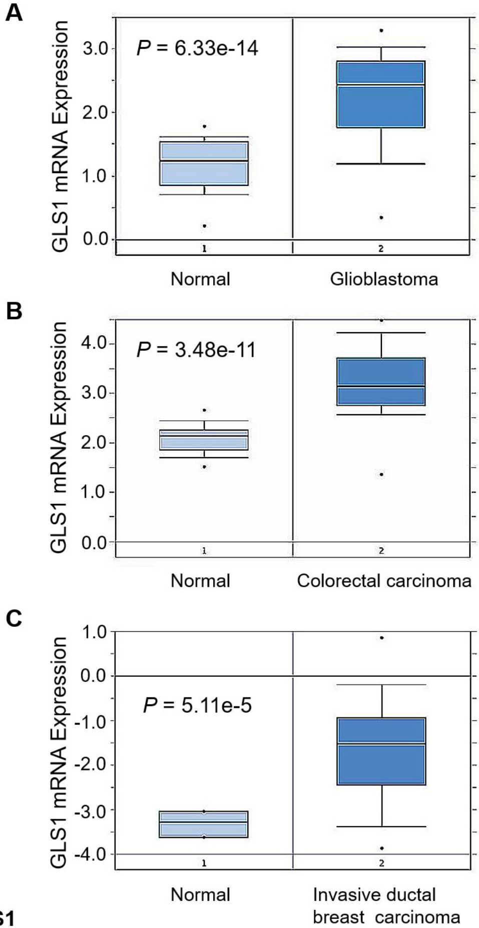
A
B
C
GLS1 mRNA Expression
P = 6.33e-14
P = 3.48e-11
P = 5.11e-5
Normal
Glioblastoma
Colorectal carcinoma
Invasive ductal breast carcinoma

Figure S1

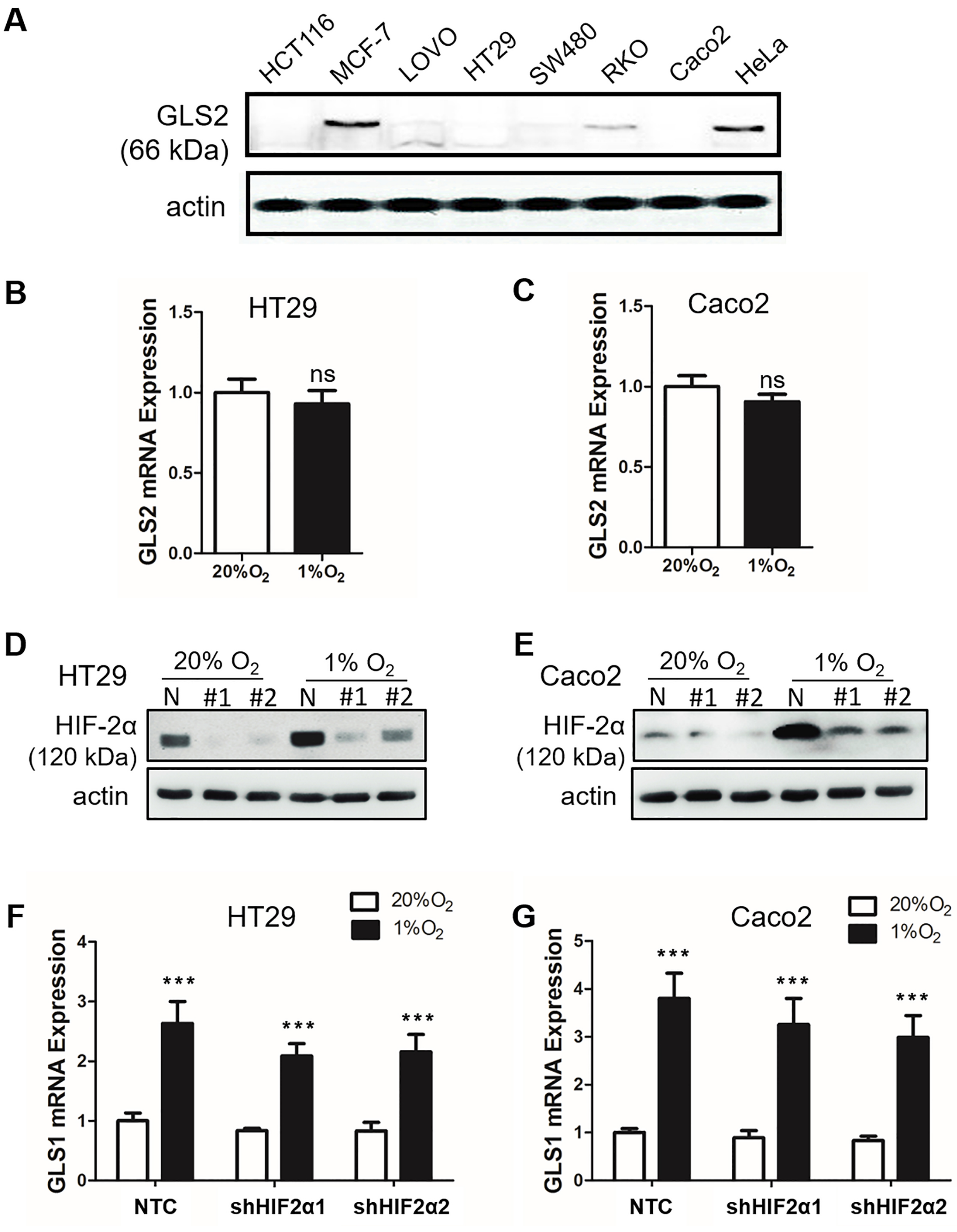

A
HCT116
MCF-7
LOVO
HT29
SW480
RKO
Caco2
HeLa
GLS2
(66 kDa)
actin
B
HT29
GLS2 mRNA Expression
1.5
1.0
0.5
0.0
ns
20%O2
1%O2
C
Caco2
GLS2 mRNA Expression
1.5
1.0
0.5
0.0
ns
20%O2
1%O2
D
HT29
20% O2
1% O2
N
#1
#2
N
#1
#2
HIF-2α
(120 kDa)
actin
E
Caco2
20% O2
1% O2
N
#1
#2
N
#1
#2
HIF-2α
(120 kDa)
actin
F
HT29
20%O2
1%O2
GLS1 mRNA Expression
4
3
2
1
0
***
***
***
NTC
shHIF2α1
shHIF2α2
G
Caco2
20%O2
1%O2
GLS1 mRNA Expression
5
4
3
2
1
0
***
***
***
NTC
shHIF2α1
shHIF2α2

Figure S2

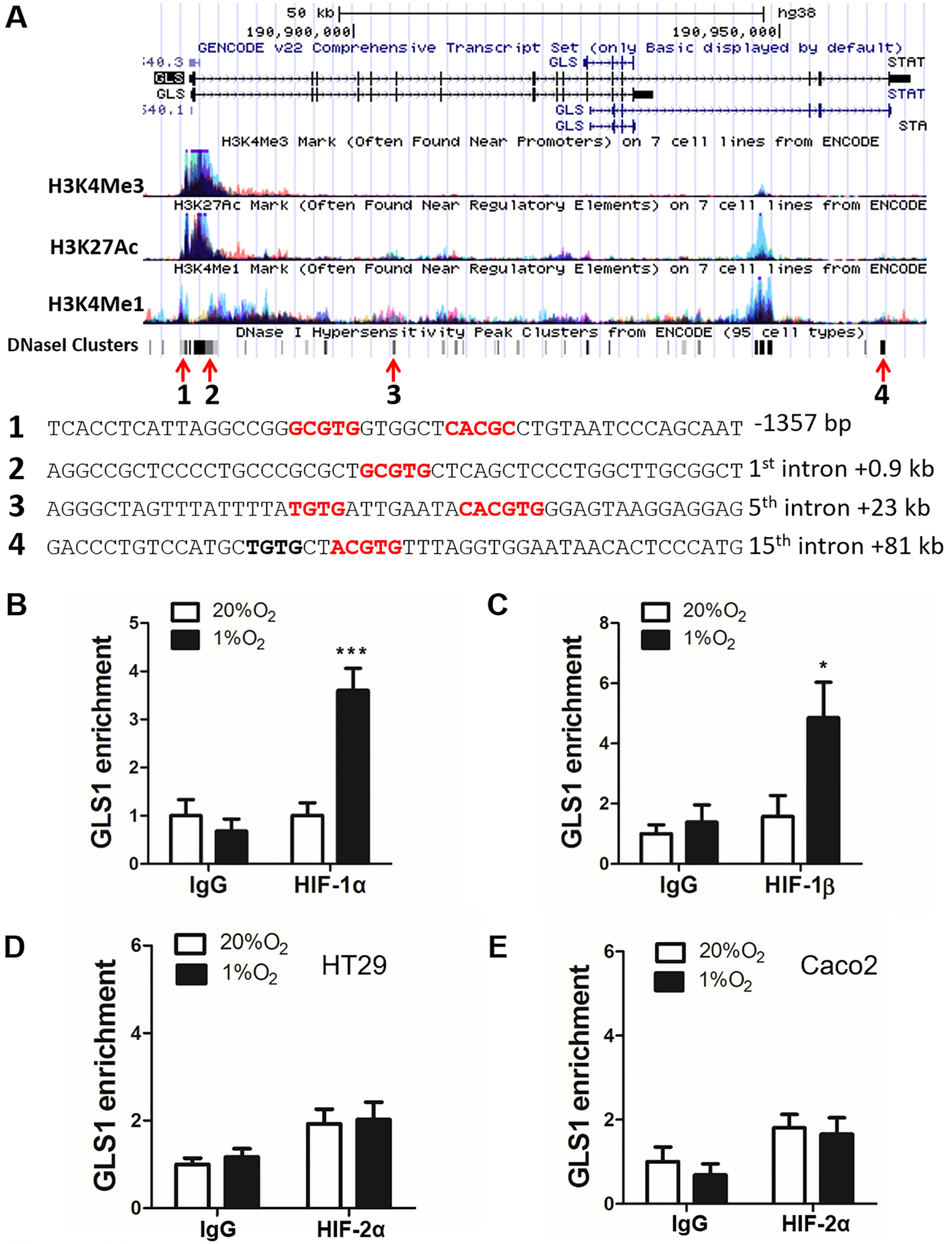
A
50 kb
hg38
190,900,000
190,950,000
GENCODE v22 Comprehensive Transcript Set (only Basic displayed by default)
GLS
STAT
H3K4Me3 Mark (Often Found Near Promoters) on 7 cell lines from ENCODE
H3K4Me3
H3K27Ac Mark (Often Found Near Regulatory Elements) on 7 cell lines from ENCODE
H3K27Ac
H3K4Me1 Mark (Often Found Near Regulatory Elements) on 7 cell lines from ENCODE
H3K4Me1
DNase I Hypersensitivity Peak Clusters from ENCODE (95 cell types)
DNaseI Clusters
1 2 3 4
1 TCACCTCATTAGGCCGGGCGTGGTGGCTCACGCCTGTAATCCCAGCAAT -1357 bp
2 AGGCCGCTCCCCTGCCCGCGCTGCGTGCTCAGCTCCCTGGCTTGCGGCT 1st intron +0.9 kb
3 AGGGCTAGTTTATTTTATGTGATTGAATACACGTGGGAGTAAGGAGGAG 5th intron +23 kb
4 GACCCTGTCCATGCTGTGCTACGTGTTTAGGTGGAATAACACTCCCATG 15th intron +81 kb
B
20%O2
1%O2
GLS1 enrichment
***
IgG
HIF-1α
C
*
HIF-1β
D
HT29
HIF-2α
E
Caco2

Figure S3

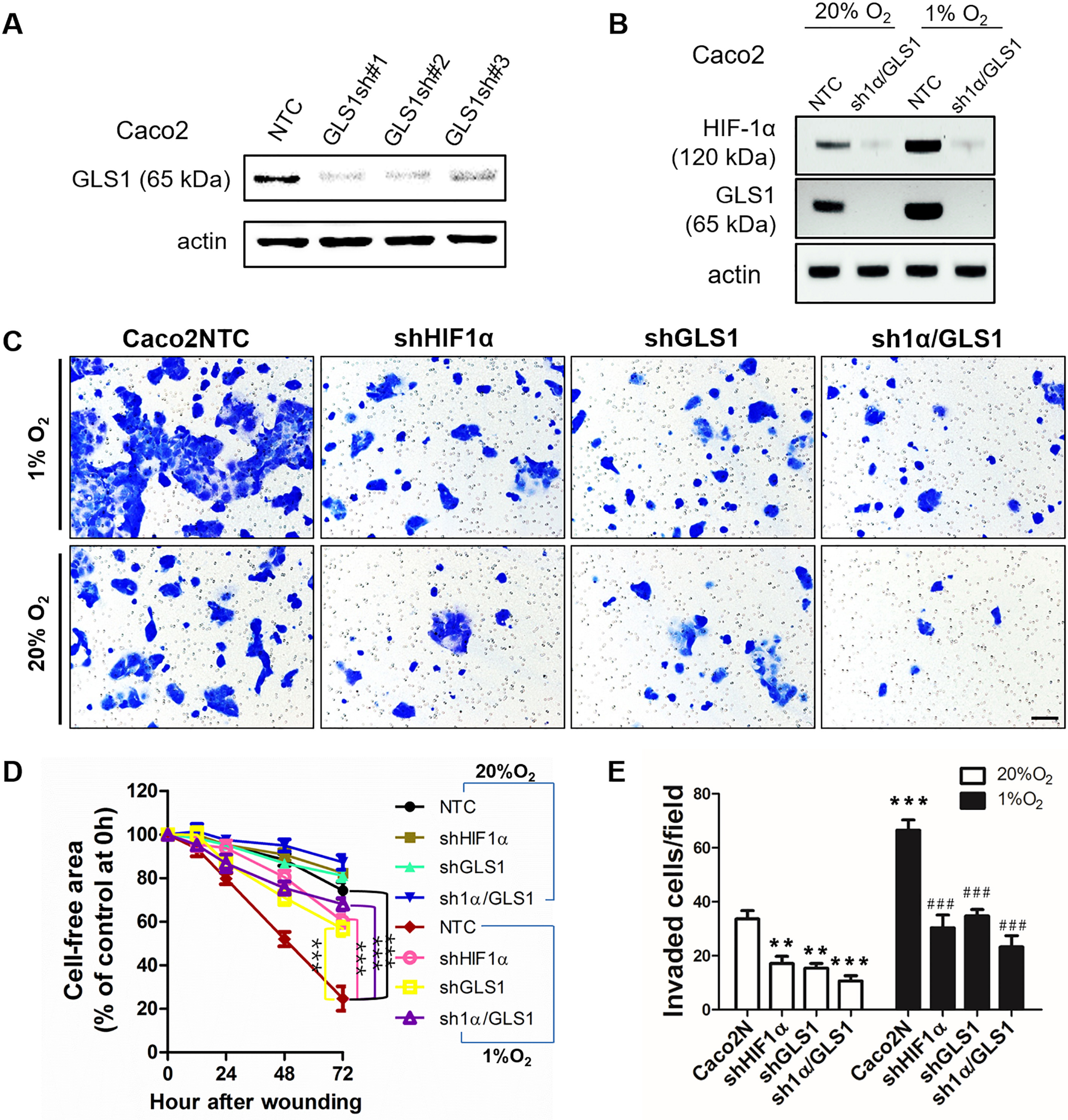

A
Caco2
NTC
GLS1sh#1
GLS1sh#2
GLS1sh#3
GLS1 (65 kDa)
actin
B
20% O2
1% O2
Caco2
NTC
sh1α/GLS1
NTC
sh1α/GLS1
HIF-1α (120 kDa)
GLS1 (65 kDa)
actin
C
Caco2NTC
shHIF1α
shGLS1
sh1α/GLS1
1% O2
20% O2
D
Cell-free area (% of control at 0h)
Hour after wounding
20%O2
1%O2
NTC
shHIF1α
shGLS1
sh1α/GLS1
E
Invaded cells/field
20%O2
1%O2
Caco2N
shHIF1α
shGLS1
sh1α/GLS1

Figure S4

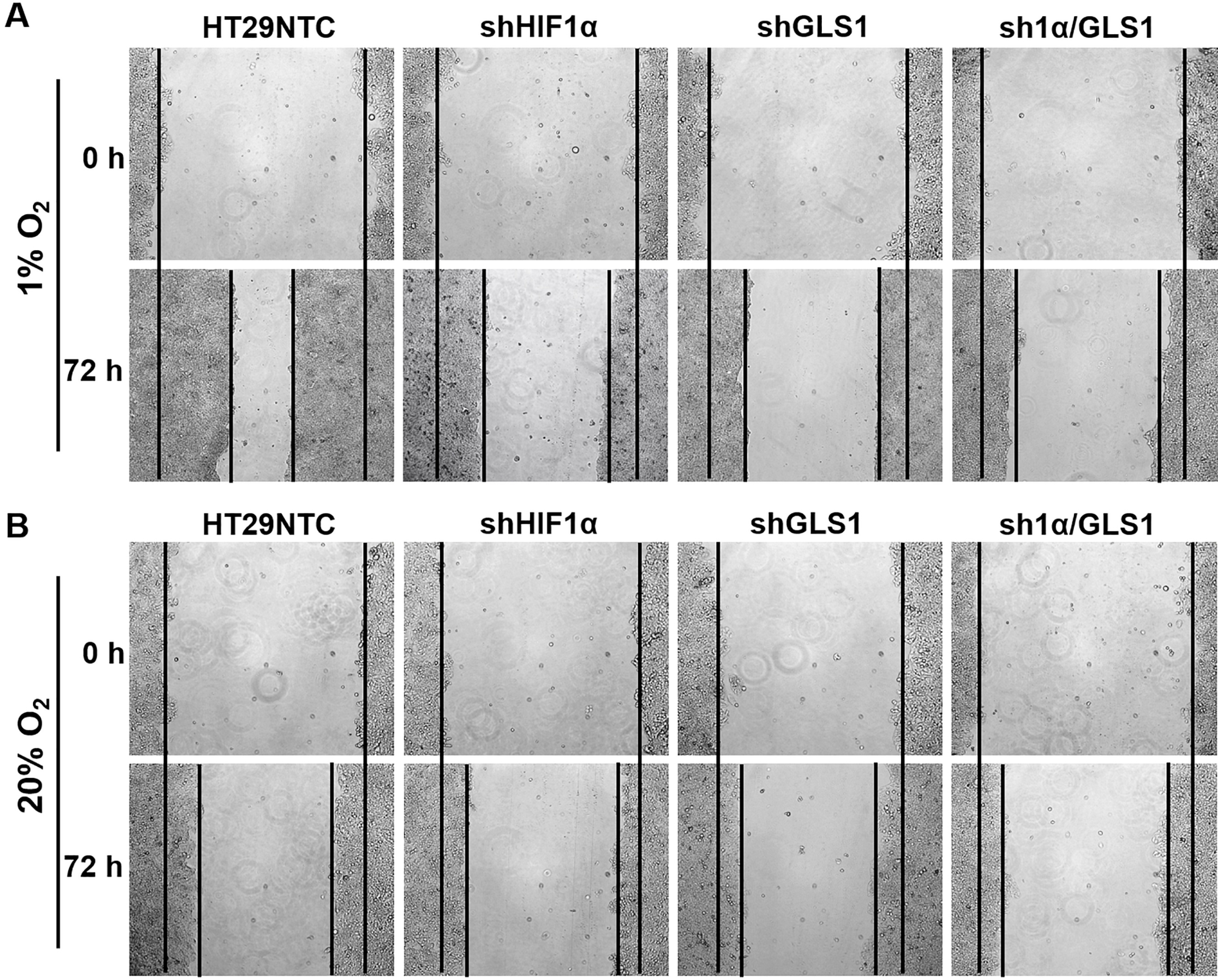
A
HT29NTC
shHIF1α
shGLS1
sh1α/GLS1
0 h
72 h
1% O2
B
HT29NTC
shHIF1α
shGLS1
sh1α/GLS1
0 h
72 h
20% O2

Figure S5

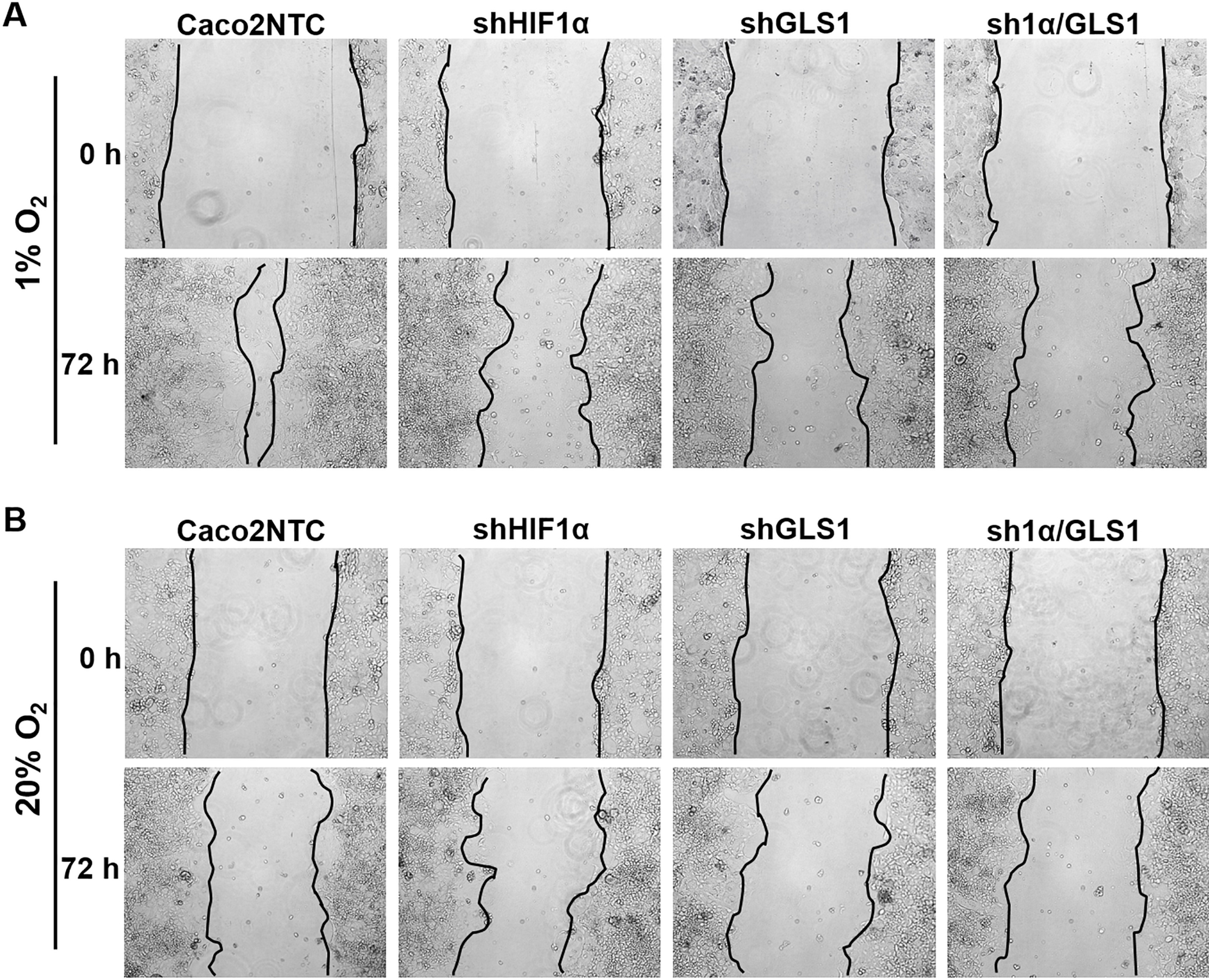
A
Caco2NTC
shHIF1α
shGLS1
sh1α/GLS1
1% O2
0 h
72 h
B
Caco2NTC
shHIF1α
shGLS1
sh1α/GLS1
20% O2
0 h
72 h

Figure S6

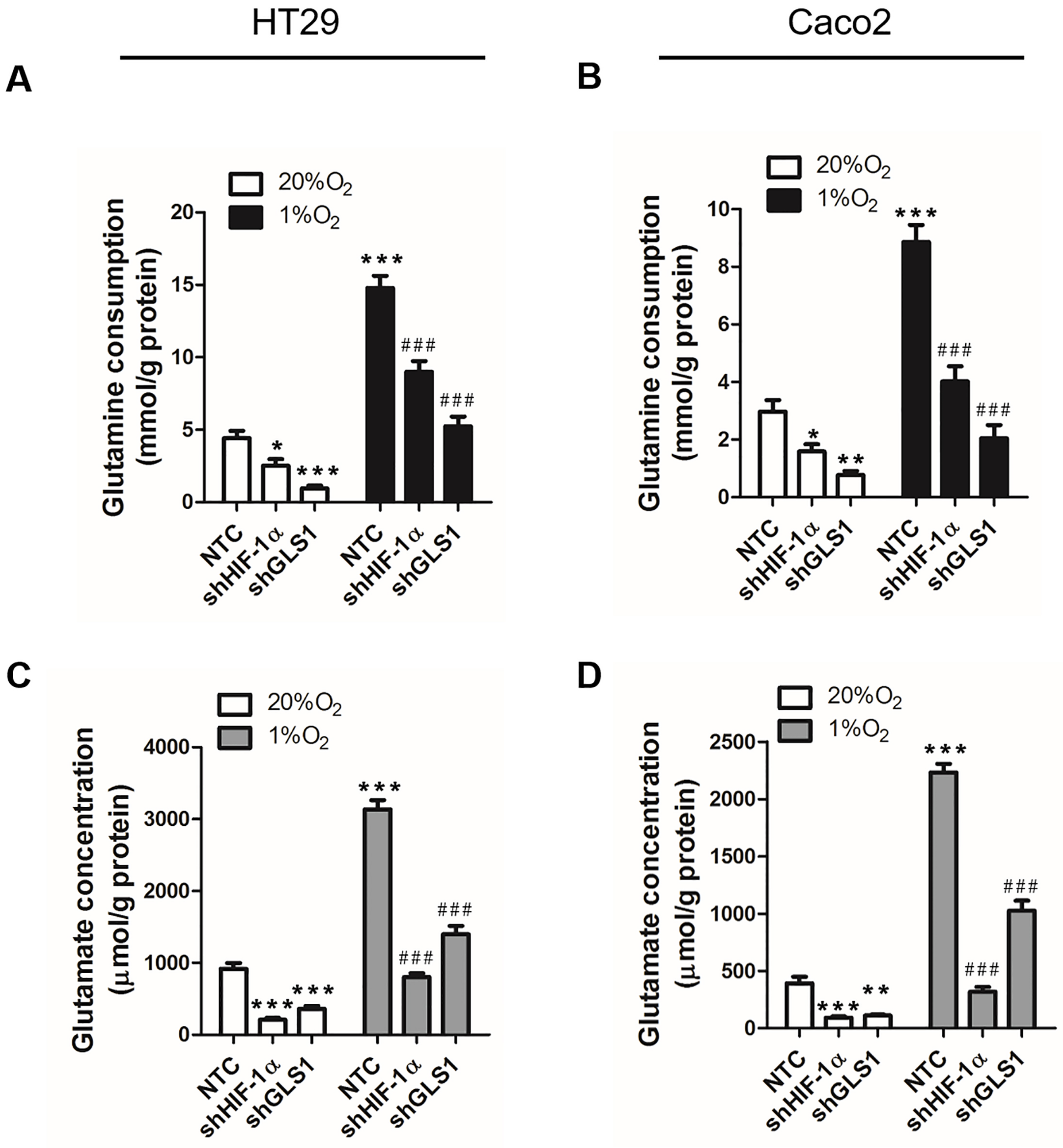
HT29
Caco2
A
B
C
D
20%O2
1%O2
Glutamine consumption (mmol/g protein)
Glutamate concentration (μmol/g protein)
NTC
shHIF-1α
shGLS1
*
**
***
###

Figure S7

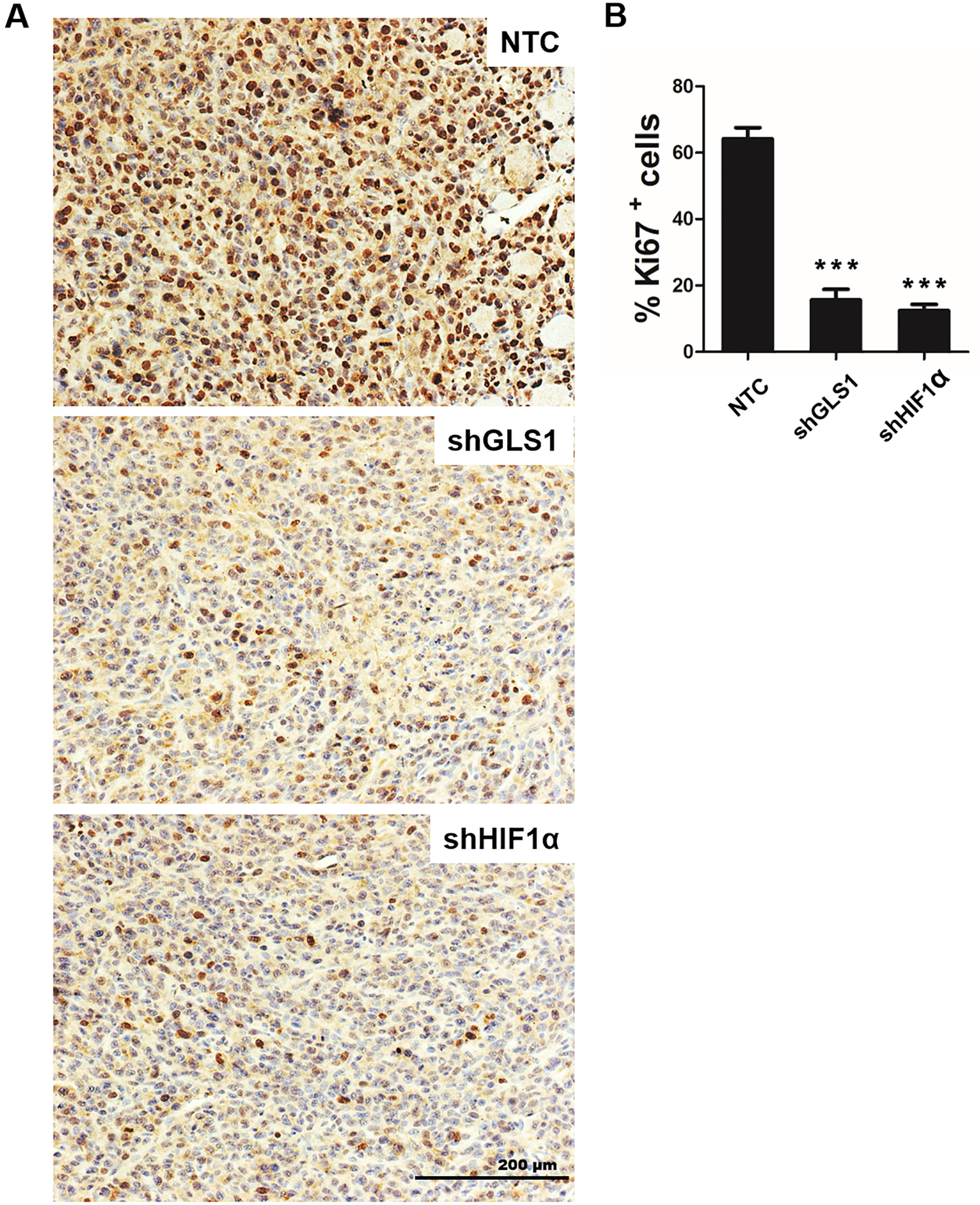
A
NTC
shGLS1
shHIF1α
200 µm
B
% Ki67 + cells
80
60
40
20
0
***
***
NTC
shGLS1
shHIF1α

Figure S8
