# Supplementary material for: Glutaminase 1 expression in colorectal cancer cells is induced by hypoxia and required for tumor growth, invasion, and metastatic colonization
Source: Cell Death Dis. 2019 Jan 17;10(2):40. doi: 10.1038/s41419-018-1291-5 (PMC6426853; doi:10.1038/s41419-018-1291-5)
Supplement: Supplementary file 3 — supplemental tables [file 41419_2018_1291_MOESM3_ESM.pdf]

Table S1 Relationship between GLS1 and GLS2 expression in tissue microarrays which contain a serial set of colorectal tissues

Variables	Total	Number of patients					
		GLS1 expression (%)		χ^2 (*P*)	GLS2 expression (%)		χ^2 (*P*)
		Low	High		Low	High	
Normal colorectal tissue	15	15 (100.0)	0 (0.0)	<0.0001	7 (46.7)	8 (53.3)	<0.0001
Inflammatory hyperplasia	17	16 (94.1)	1 (5.9)		6 (35.2)	11 (64.7)	
Colorectal adenoma	9	9 (100.0)	0 (0.0)		9 (100.0)	0 (0.0)	
Colorectal adenocarcinoma	39	13 (33.3)	26 (66.7)		39 (100.0)	0 (0.0)	

Table S2 Relationship between clinicopathological parameters of colon cancer cases and expression of GLS1

**Variables**	Total	Number of patients (%)		Chi-square test
		GLS1 low expression	GLS1 high expression	*P* value
**Age (year)**				
<60	38	13 (34.2)	25 (65.8)	0.052[a]
≥60	47	26 (55.3)	21 (44.7)	
**Gender**				
Male	50	21 (42.0)	29 (58.0)	0.391[a]
Female	35	18 (51.4)	17 (48.6)	
**Lymph node metastasis**				
Present	24	4 (16.7)	20 (83.3)	0.001[b]
Absent	61	35 (57.4)	26 (42.6)	
**Grade**				
well and moderately differentiated	60	31 (51.7)	29 (48.3)	0.097[a]
poorly and non-differentiated	25	8 (32.0)	17 (68.0)	
**Clinical stage**				
early stage	51	34 (66.7)	17 (33.3)	<0.001[b]
advanced stage	34	5 (17.4)	29 (85.3)	

Median values were used as cut-off points for definition of age subgroups
a, Pearson Chi-Square Test; b, Fisher's Exact Test

Table S3 *GLS1* gene expression is correlated with the expression of known HIF target genes in colorectal carcinomas

Category	*HIF targets*										*Non-HIF target*
**GENE**	***VEGFA***	***PDGFB***	***LOX***	***CXCR3***	***ANGPTL4***	***L1CAM***	***SLC2A1***	***P4HA1***	***P4HA2***	***MET***	***RPL4***
GLS1	***	**	*	**	***	***	**	*n.s.*	***	***	*n.s.*
PDGFB	**	—	***	**	***	***	***	***	*	*n.s.*	*n.s.*
LOX	*n.s.*	***	—	**	***	***	***	***	***	*n.s.*	*n.s.*
MET	***	*n.s.*	*n.s.*	*n.s.*	**	***	***	**	*n.s.*	—	*n.s.*

*p <0.05, **p <0.01, ***p <0.001, n.s., not significant, Pearson's correlation test of microarray data from The Cancer Genome Atlas (TCGA) dataset of 433 colorectal carcinomas.

Table S4. Sequences of Oligonucleotide Primers Used for RT-qPCR and ChIP, Related to Methods.

Primers used in qRT-PCR assays	Sequence
Human-GLS1-FWD	tctacaggattgcgaacgtct
Human-GLS1-REV	ctttgtctagcatgacaccatct
Human-GLS2-FWD	atcagaaagtggcatgctgt
Human-GLS2-REV	gcctttagtgcagtggtgaa
Human-HK2-FWD	ccagttcattcacatcatcag
Human-HK2-REV	cttacacgaggtcacatagc
Human and Mouse-18S-FWD	cggcgacgacccattcgaac
Human and Mouse-18S-REV	gaatcgaaccctgattccccgtc
Human-18S-FWD	gaggatgaggtggaacgtgt
Human-18S-REV	agaagtgacgcagccctcta
**Primers used in ChIP assays**	**Sequence**
PDK1-HRE-FWD	cgcgtttggattccgtg
PDK1-HRE-REV	ccagttataatctgccttccctattatc
GLS1-HRE-FWD	tgcacctgacatacaccctt
GLS1-HRE-REV	ctggtttcgagggatctgca
